# The Parkinson’s disease-associated gene ITPKB protects against α-synuclein aggregation by regulating ER-to-mitochondria calcium release

**DOI:** 10.1073/pnas.2006476118

**Published:** 2020-12-21

**Authors:** Daniel J. Apicco, Evgeny Shlevkov, Catherine L. Nezich, David T. Tran, Edward Guilmette, Justin W. Nicholatos, Collin M. Bantle, Yi Chen, Kelly E. Glajch, Neeta A. Abraham, Lan T. Dang, G. Campbell Kaynor, Ellen A. Tsai, Khanh-Dung H. Nguyen, Joost Groot, YuTing Liu, Andreas Weihofen, Jessica A. Hurt, Heiko Runz, Warren D. Hirst

**Affiliations:** ^a^Neurodegenerative Diseases Research Unit, Biogen, Cambridge, MA 02142;; ^b^Biogen Postdoctoral Scientist Program, Biogen, Cambridge, MA 02142;; ^c^Gene Therapy Accelerator Unit, Biotherapeutics and Medicinal Sciences, Biogen, Cambridge, MA 02142;; ^d^Multiple Sclerosis and Neurorepair Research Unit, Biogen, Cambridge, MA 02142;; ^e^Human Genetics, Translational Biology, Biogen, Cambridge, MA 02142;; ^f^Genome Technologies and Scientific Computing, Translational Biology, Biogen, Cambridge, MA 02142;; ^g^Biologics Drug Discovery, Biotherapeutics and Medicinal Sciences, Biogen, Cambridge, MA 02142;; ^h^Human Target Validation Core, Translational Biology, Biogen, Cambridge, MA 02142

**Keywords:** Parkinson’s disease, genetics, α-synuclein, calcium signaling, mitochondria

## Abstract

Inositol-1,4,5-triphosphate (IP_3_) kinase B (ITPKB) is a ubiquitously expressed lipid kinase that inactivates IP_3_, a secondary messenger that stimulates calcium release from the endoplasmic reticulum (ER). Genome-wide association studies have identified common variants in the ITPKB gene locus associated with reduced risk of sporadic Parkinson’s disease (PD). Here, we investigate whether ITPKB activity or expression level impacts PD phenotypes in cellular and animal models. In primary neurons, knockdown or pharmacological inhibition of ITPKB increased levels of phosphorylated, insoluble α-synuclein pathology following treatment with α-synuclein preformed fibrils (PFFs). Conversely, ITPKB overexpression reduced PFF-induced α-synuclein aggregation. We also demonstrate that ITPKB inhibition or knockdown increases intracellular calcium levels in neurons, leading to an accumulation of calcium in mitochondria that increases respiration and inhibits the initiation of autophagy, suggesting that ITPKB regulates α-synuclein pathology by inhibiting ER-to-mitochondria calcium transport. Furthermore, the effects of ITPKB on mitochondrial calcium and respiration were prevented by pretreatment with pharmacological inhibitors of the mitochondrial calcium uniporter complex, which was also sufficient to reduce α-synuclein pathology in PFF-treated neurons. Taken together, these results identify ITPKB as a negative regulator of α-synuclein aggregation and highlight modulation of ER-to-mitochondria calcium flux as a therapeutic strategy for the treatment of sporadic PD.

## Significance

Parkinson’s disease (PD) is the second most prevalent neurodegenerative disease of aging, affecting approximately 10 million patients worldwide with no approved therapies to modify progression of disease. Further understanding of the cellular mechanisms contributing to the development of PD is necessary to discover therapies. Here, we characterize the role of a recently identified GWAS hit for sporadic PD, ITPKB, in the aggregation of α-synuclein, the primary pathological feature of disease. These results identify inhibition of inositol-1,4,5,-triphosphate (IP_3_)-mediated ER-to-mitochondria calcium release as a potential therapeutic approach for reducing neuropathology in PD.

Parkinson’s disease (PD) is a progressive neurodegenerative disorder characterized by a variety of motor symptoms (including unbalanced gait, resting tremor, and bradykinesia) that are accompanied by psychosis and dementia at later stages of disease. The onset of motor symptoms is largely caused by the selective loss of dopaminergic neurons in the substantia nigra pars compacta (SNpc) and the corresponding depletion of dopamine innervation in the striatum. Although the cause of neuron loss is unknown, the hallmark pathological feature of PD is the presence of intraneuronal inclusions composed of misfolded and fibrillar α-synuclein (α-syn) in the neurites and soma, termed Lewy neurites and Lewy bodies, respectively (1). Protein-coding single-nucleotide polymorphisms (SNPs), duplications, and triplications in the gene encoding α-syn (SNCA) all cause early-onset, familial forms of PD and result in accelerated aggregation of α-syn protein into insoluble, fibrillar aggregates (1, 2). Recent evidence suggests that these aggregates can spread from cell to cell, leading to the propagation of pathology to neuroanatomically connected brain regions (3, 4). Therefore, therapeutic approaches that reduce the aggregation or spreading of pathological α-syn species represent potential disease-modifying therapies for PD.

In addition to SNCA, mutations in several other genes have been identified that cause rare, familial forms of PD. These genes are primarily involved in the autophagic clearance of intracellular aggregates or damaged organelles, especially mitochondria (5–8). Genome-wide association studies (GWAS) have also identified common SNPs in several genes related to endolysosomal function, such as GBA and LRRK2, that are associated with increased risk of PD (8–11). GBA and LRRK2 mutations have been shown to affect lysosomal and mitochondrial phenotypes (12–14), which contribute to the accumulation of PD-like neuropathology in mouse models, primary neurons, and human iPSC-derived cells (15–18). These findings highlight the role of the lysosomal and mitochondria quality control pathways in PD and demonstrate that perturbations in these pathways are sufficient to increase α-syn aggregation. Despite this, the etiology of sporadic PD is not fully understood, and the specific genes and pathways that are tractable for therapeutic modulation remain elusive. Therefore, the discovery of new genes associated with sporadic PD may be critical for both understanding disease pathogenesis and identifying novel therapeutic approaches.

Recently, Chang et al. conducted a GWAS analysis that identified 17 novel gene loci significantly associated with sporadic PD in a European population including more than 26,000 patients across three independent cohorts (19). The lead GWAS SNP in the novel 1q42 locus was rs4653767. This is an intronic SNP in the gene encoding inositol-1,4,5-triphosphate kinase B (ITPKB) and produces a thymine-to-cytosine nucleotide substitution that is protective against developing PD (odds ratio [OR] = 0.92, *P* = 2.4 × 10^−10^). A follow up meta-analysis study of 37,688 PD patients, which included the discovery cohort, and 18,618 proxy cases strengthened the GWAS finding at this locus (OR = 0.92, *P* = 1.4 × 10^−15^). Furthermore, this locus was still significant when the analysis was performed on only the new independent cases and proxy cases (OR = 0.92, *P* = 2.8 × 10^−5^) (20). The rs4653767-C allele is present in similar frequencies across populations (27% in non-Finnish European and 29% in East Asian populations) and was found to have the same direction of effect (OR = 0.87, *P* = 0.016) in a targeted replication study of the European PD loci in an East Asian cohort (21). ITPKB is also highly expressed in several brain regions related to PD, including the SNpc, striatum, and cerebral cortex (22).

ITPKB is one of three ubiquitously expressed kinases known to phosphorylate inositol-1,4,5-triphosphate (IP_3_), an intracellular messenger produced from phosphatidylinositol-4,5-bisphosphate by phospholipase C (23, 24). IP_3_ binds to IP_3_ receptors (IP_3_Rs) in the endoplasmic reticulum (ER) to stimulate the release of calcium ions from the ER into the cytosol to mediate various downstream signaling pathways. IP_3_ kinases (ITPKA, ITPKB, and ITPKC) add a fourth phosphate group to IP_3_ producing inositol-1,3,4,5-tetrakisphosphate (IP_4_), which has no activity on IP_3_Rs. Thus, IP_3_ kinases negatively regulate IP_3_-mediated calcium release from the ER. While the role of this pathway in peripheral cell types under normal physiological conditions is well understood (25, 26), whether ITPKB is involved in the pathogenesis of PD is unknown. Here, we investigate whether the modulation of ITPKB expression or kinase activity impacts the accumulation of α-syn pathology in cellular models of PD.

## Results

### ITPKB Inhibition Increases α-Syn Pathology Induced by Preformed Fibrils.

ITPKB has previously been reported to be the most abundantly expressed IP_3_ kinase in the central nervous system across several brain regions and cell types, including neurons (27). In contrast, ITPKA is expressed primarily in neurons, while ITPKC expression is restricted to glia (27). qPCR analysis of ITPKA, ITPKB, and ITPKC expression in primary mouse cortical neurons (referred to as “primary neurons” or simply “neurons” in the rest of this manuscript), adult mouse brain tissue, and human iPSC-derived neurons and astrocytes confirmed that ITPKB is the most abundantly expressed IP_3_ kinase compared to ITPKA and ITPKC (*SI Appendix*, Fig. S1 *A*–*D*). An analysis of publicly available brain expression (*SI Appendix*, Fig. S1*E*) and single-cell RNA-sequencing databases confirmed ITPKB expression in several neuronal subtypes in the human and mouse brain as well as in astrocytes and oligodendrocytes (*SI Appendix*, Fig. S1 *F* and *G*). Immunoblot analysis confirmed endogenous expression of the ITPKB protein in primary neurons, which could be knocked down by transduction with lentiviruses (LVs) expressing a short hairpin RNA (shRNA) sequence targeting murine ITPKB (mITPKB shRNA LV; *SI Appendix*, Fig. S1*H*). Intracellular calcium levels were also increased in neurons following treatment with GNF362, an ATP-competitive pan-ITPK inhibitor with low nanomolar affinity (25, 28), confirming that IP_3_-mediated ER calcium release is a biologically relevant cellular pathway in neurons (*SI Appendix*, Fig. S1*I*). We therefore concluded that mouse primary neurons are a relevant model system for studying ITPKB function.

We first investigated whether ITPKB activity or expression level impacted α-syn pathology in mouse neurons. Various groups have demonstrated that treatment with recombinant α-syn preformed fibrils (PFFs) or α-syn aggregates derived from PD brain tissue are able to seed de novo α-syn aggregates in primary neurons that replicate the biochemical properties of α-syn aggregates found in PD patients (29–31), which includes the accumulation of insoluble and high-molecular-weight (mw) α-syn species that are aberrantly phosphorylated at serine 129 (pS129). Thus, we investigated whether inhibition of ITPKB in neurons impacted the accumulation of pS129 α-syn induced by treatment with PFFs.

**Fig. 1.**
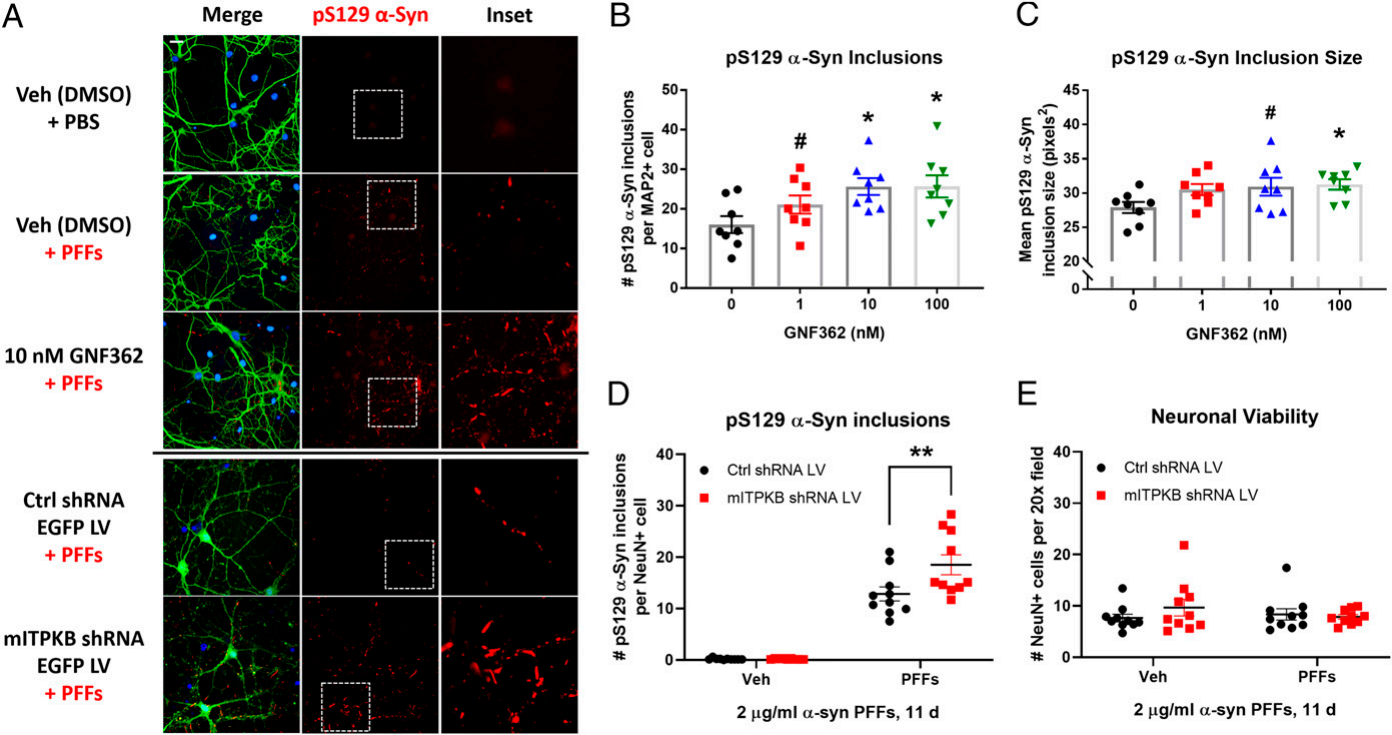
ITPKB inhibition increases α-syn pathology induced by preformed fibrils. (*A*) Representative images of DIV 20 neurons immunostained for pS129 α-syn (red), the neuronal marker MAP2 (*Top*, green), and DAPI (blue); LV-transduced cells are labeled with EGFP expressed from the same virus as the shRNAs (*Bottom*). Scale bar, 10 µm. (*B*) Quantification of the number of pS129 α-syn inclusions per MAP2-positive cell in *A*. ^#^*P* = 0.1501; *P = 0.0197 (10 nM); *P = 0.0193 (100 nM) by one-way ANOVA with Dunnett’s post hoc test (versus the 0 nM treatment group); *n* = 8 wells/group. (*C*) Quantification of the mean pS129 α-syn inclusion size in GNF362-treated cultures in *A*. ^#^*P* = 0.0806; *P = 0.0473 by one-way ANOVA with Dunnett’s post hoc test (versus the 0 nM treatment group); *n* = 8 wells/group. (*D*) Quantification of the number of pS129 α-syn inclusions per NeuN-positive cell in cells transduced with control (Ctrl) or mITPKB shRNA LV from *A*. **P = 0.0035 by two-way ANOVA with Sidak’s post hoc test; *n* = 10 wells/group. (*E*) Quantification of the number of NeuN-positive cells per 20× field in cells transduced with Ctrl or mITPKB shRNA LV from *A*. Note that no toxicity was observed for either the mITPKB shRNA LV or treatment with PFFs. All figures shown are representative examples of the experiments repeated at least three times in independent cultures of primary neurons with different plate layouts. All bar graphs represent the mean ± SEM.

As expected, neurons treated with 2 µg/mL α-syn PFFs for 11 d exhibited a significant accumulation of pS129 α-syn, as determined by immunocytochemical (ICC) analysis; treatment with vehicle (phosphate-buffered saline [PBS]) or 2 µg/mL α-syn monomer had no effect (*SI Appendix*, Fig. S2 *A*–*E*). Compared to control cells, neurons treated with GNF362 exhibited a dose-dependent increase in the number and size of pS129 α-syn–positive inclusions induced by PFFs (Fig. 1 *A*–*C*), suggesting that inhibition of IP_3_ kinase activity increases α-syn pathology. Similarly, neurons transduced with mITPKB shRNA LV exhibited an increase in the number of pS129 α-syn inclusions per neuron (Fig. 1*D*), confirming that specific knockdown of ITPKB is sufficient to increase PFF-induced α-syn pathology. Despite the robust induction of pathology in neurons treated with PFFs, no significant neuron loss was observed at 20 d in vitro (DIV) (Fig. 1*E*), consistent with previously published reports (32).

We next investigated whether ITPKB inhibition increased α-syn pathology in human neurons differentiated from induced pluripotent stem cells (iPSCs). We used iPSCs that were reprogrammed from fibroblasts isolated from a PD patient harboring an SNCA gene triplication and stably transduced with a doxycycline-inducible neuroligin 2 (NGN2) LV, which, upon doxycycline treatment, is sufficient to directly induce differentiation of iPSCs to functional excitatory neurons in less than 2 wk (33). We chose NGN2 neurons because these cells have been previously reported to develop pS129 α-syn pathology in response to α-syn PFF treatment (18). NGN2 LV-transduced iPSCs were differentiated into neurons in the presence of 1 µg/mL doxycycline for 11 d followed by treatment with 5 µg/mL murine PFFs and GNF362 for 10 d (*SI Appendix*, Fig. S3*A*). Consistent with the results in primary neurons, treatment with 100 nM GNF362 increased the number of pS129 α-syn inclusions in human iPSC-derived neurons (*SI Appendix*, Fig. S3 *B*–*E*).

**Fig. 2.**
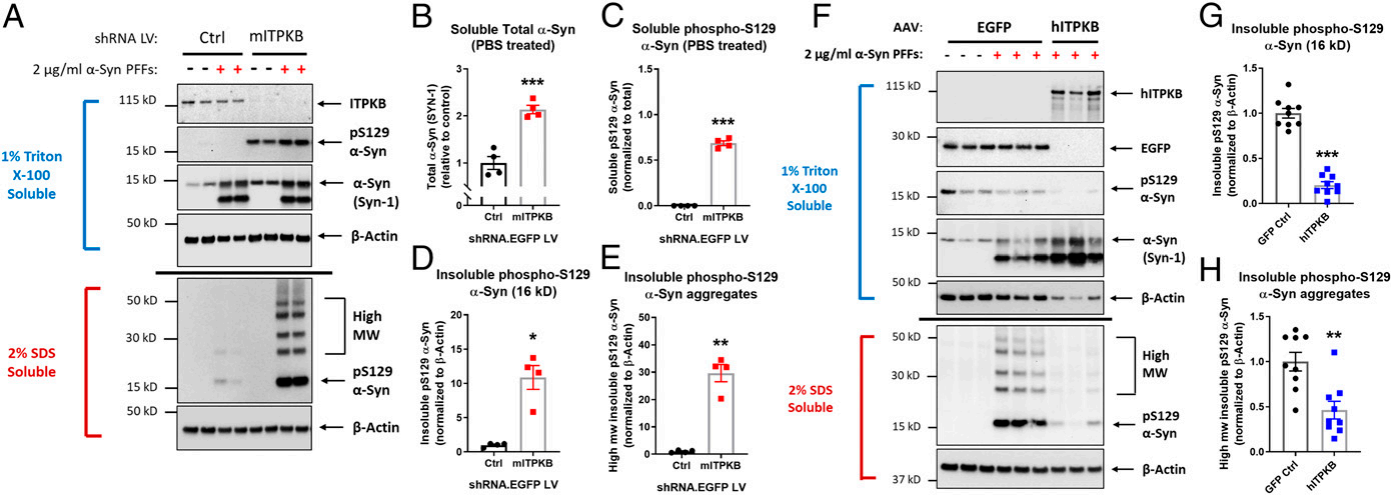
ITPKB expression level regulates the accumulation of insoluble α-syn aggregates. (*A*) A representative immunoblot of primary neurons transduced with control (Ctrl) or mITPKB shRNA LV and treated with 2 µg/mL murine α-syn PFFs for 11 d followed by biochemical fractionation into 1% Triton X-100 soluble (*Upper*, blue) and insoluble (2% SDS soluble, *Lower*, red) fractions. The samples were probed for levels of ITPKB, β-Actin (loading control), total α-syn (SYN-1), and pS129 α-syn. (*B*, *C*) Quantification of relative total (SYN-1) and pS129 α-syn in the soluble fraction of neurons transduced with Ctrl or mITPKB shRNA LV. Note that the SYN-1 antibody also detects murine PFFs present in each sample; therefore, total and pS129 α-syn pathology in the soluble fraction was only quantified for the samples not treated with PFFs (PBS treated). ***P < 0.001 by unpaired Student’s *t* test; *n* = 4 wells/group. (*D*, *E*) Quantification of relative monomeric (16 kDa, *D*) and sum of the mutimeric (high-mw pS129 α-syn aggregates, *E*) pS129 α-syn levels present in the 2% SDS fraction of neurons transduced with Ctrl or mITPKB shRNA LV and treated with PFFs. *P = 0.0105; **P = 0.0028 by Student’s *t* test; *n* = 4 wells/group. (*F*) Representative immunoblots of neurons transduced with GFP (Ctrl) or hITPKB expressing AAVs and treated with 2 µg/mL murine α-syn PFFs for 11 d followed by biochemical fractionation. The samples were probed for levels of ITPKB, β-Actin (loading control), and total (SYN-1) and pS129 α-syn. (*G*, *H*) Quantification of the relative levels of both the monomeric (16 kDa, *G*) and mutimeric (high-mw, *H*) pS129 α-syn bands in the 2% SDS fraction of PFF-treated neurons from *F*. ***P < 0.001; **P = 0.0016 by Student’s *t* test; *n* = 9 wells/group. All figures shown are representative examples of experiments repeated at least three times in independent cultures of primary neurons with different plate layouts. The bar graphs represent the mean ± SEM.

In order to further investigate whether ITPKB inhibition impacts the solubility properties of α-syn aggregates, we examined PFF treated neurons transduced with control shRNA LV and mITPKB shRNA LV by biochemical fractionation. Knockdown of ITPKB increased the levels of total and pS129 α-syn in both the soluble (“1% Triton X-100 soluble”) and insoluble (“2% SDS [sodium dodecyl sulfate] soluble”) fractions (Fig. 2 *A*–*E*). The increase in pS129 α-syn accumulation was particularly notable in the 2% SDS fraction, where there was nearly a 30-fold increase in high-mw α-syn species in mITPKB shRNA LV-transduced neurons (Fig. 2*E*). Consistent with this, 11-d treatment with GNF362 also increased the level of pS129 α-syn in the insoluble fraction of primary neurons (*SI Appendix*, Fig. S4 *A* and *B*). We next produced adeno-associated viruses (AAVs) expressing human ITPKB with a C terminus Flag tag (hITPKB-Flag) to determine if hITPKB overexpression could reduce α-syn pathology. Consistent with the results from mITPKB shRNA LV-transduced neurons (Fig. 2 *A*–*E*), cells transduced with hITPKB-Flag AAV exhibited reduced levels of insoluble pS129 α-syn following treatment with PFFs compared to control-transduced cells (Fig. 2 *F*–*H*). This reduction was observed for both monomeric (16-kDa band; Fig. 2*G*) and high-mw (Fig. 2*H*) pS129 α-syn bands. ITPKB overexpression by hITPKB-Flag AAV also reduced the number of pS129 α-syn inclusions per neuron induced by PFF treatment by ICC analysis (*SI Appendix*, Fig. S5 *A* and *B*). Taken together, these results demonstrate that ITPKB activity and expression level modulates α-syn aggregation in neurons.

### ITPKB Inhibition Increases Calcium Accumulation in Mitochondria.

**Fig. 3.**
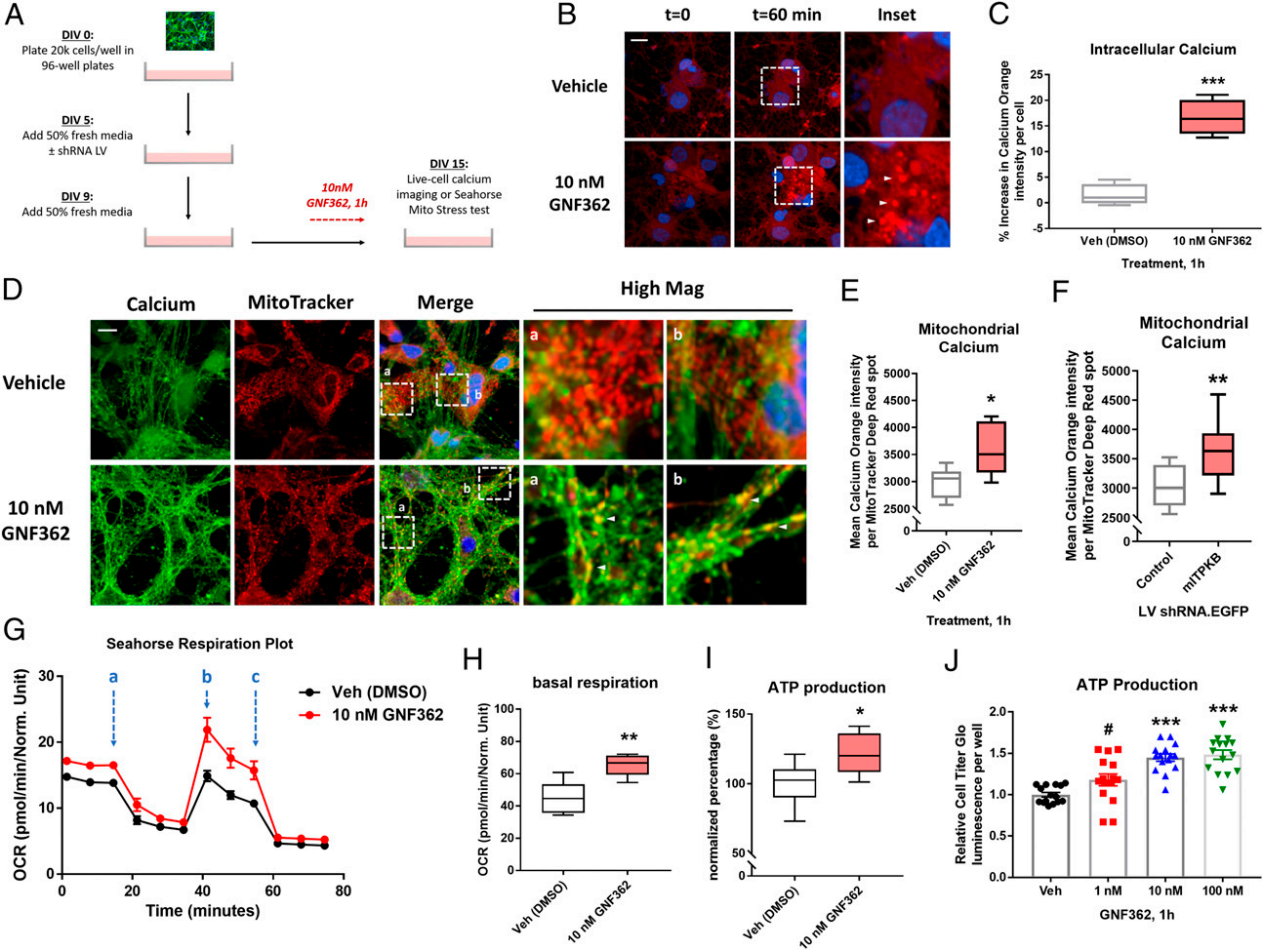
ITPKB inhibition increases mitochondrial calcium levels and ATP production. (*A*) A diagram of experimental design for live-cell calcium imaging and respiration experiments in primary neurons treated with GNF362 or transduced with LVs. (*B*) Representative images of Calcium Orange AM dye fluorescence intensity following 1 h treatment with 10 nM GNF362. (*C*) Quantification of the mean percent increase in Calcium Orange AM dye fluorescence intensity per DAPI+ cells at t = 60 min compared to t = 0. ***P < 0.001 by unpaired Mann–Whitney *U* test; *n* = 6 wells/group. (*D*) Representative images of Calcium Orange AM dye (shown in green to highlight colocalization) and MitoTracker Deep Red (red) fluorescence in DIV 15 cultures treated with 10 nM GNF362 for 1 h. (*E*) Quantification of mean Calcium Orange AM fluorescence intensity per MitoTracker Deep Red–positive spot in *D*. *P = 0.0411 by unpaired Mann–Whitney *U* test; *n* = 6 wells/group. (*F*) Quantification of mean Calcium Orange AM fluorescence intensity per MitoTracker Deep Red–positive spot in cultures transduced with control or mITPKB shRNA LV. **P = 0.0036 by unpaired Mann–Whitney *U* test; *n* = 12 wells/group. (*G*) A representative respiration plot of cultures analyzed by the Seahorse XF Mito Stress Test following pretreatment with vehicle (DMSO, black line) or 10 nM GNF362 (red line). The OCR was measured over time starting at baseline and following sequential treatment with 1 µM oligomycin (*A*), 1.5 µM FCCP (*B*), and 0.5 µM antimycin/rotenone (*C*). (*H*–*I*) Quantification of baseline OCR (*H*) and total ATP production (*I*) from *G*. **P = 0.0043; *P = 0.0118 by two-tailed Mann–Whitney *U* test; *n* = 6 wells/group. All box and whisker plots represent median ± interquartile range (box) from minimum to maximum (whiskers). (*J*) The total cellular ATP levels measured by relative Cell Titer Glo luminescence per well following 1 h treatment with GNF362. ***P < 0.001; ^#^*P* = 0.0853 by one-way ANOVA with Tukey’s post hoc test; *n* = 15 wells/group. The bar graphs represent mean ± SEM. All Seahorse respiration experiments were repeated at least three times in independent cultures of primary neurons with different plate layouts (*SI Appendix*, Fig. S6 *I* and *J*).

Given the significant effect of ITPKB inhibition on α-syn pathology, we decided to investigate the cellular consequences of ITPKB inhibition in neurons. Since treatment with the IP_3_ kinase inhibitor GNF362 increased the intracellular levels of calcium in neurons (*SI Appendix*, Fig. S1*I*), we investigated whether this increase occurred uniformly throughout the cytosol or locally in specific subcellular compartments. Neurons were preincubated with 2 µM Calcium Orange AM dye and then treated with 10 nM GNF362 followed by live-cell imaging (Fig. 3 *A* and *B*); Calcium Orange AM dye has a narrow excitation/emission spectrum that allows for multiplexing with other fluorophores, including green fluorescent protein (GFP) and far red dyes (34). Neurons exhibited a detectable increase in mean Calcium Orange AM fluorescence intensity as early as 10 min following treatment with GNF362, which peaked at approximately a 20% increase after 1 h (Fig. 3 *B* and *C*). While the mean Calcium Orange AM fluorescence intensity increased in the cytosol of neurons treated with GNF362, visual inspection of the images revealed that the increase was not evenly distributed throughout the entire cytoplasm but rather accumulated in bright puncta of intense fluorescence intensity (Fig. 3*B*, arrows). Since mitochondria have previously been reported as the predominant buffering organelle for transient increases in intracellular calcium levels, especially in neurites and axons (35), we investigated whether these Calcium Orange AM puncta colocalized with mitochondria. This possibility seemed especially plausible due to the high concentration of IP_3_Rs at sites of ER–mitochondria contact, known as mitochondria-associated membranes (MAMs), which serve to facilitate the direct transfer of calcium from ER stores to mitochondria and other organelles (36–38). To test this hypothesis, neurons were preincubated with 2 µM Calcium Orange AM and either 5 nM MitoTracker Deep Red or LysoTracker Deep Red dyes to label mitochondria and lysosomes, respectively, followed by treatment with 10 nM GNF362 or vehicle (DMSO) for 1 h. As expected, GNF362 treatment produced an increase in overall intracellular Calcium Orange AM fluorescence intensity, which was most concentrated in puncta that strongly colocalized with MitoTracker Deep Red spots, especially in neuronal processes (Fig. 3*D*, arrows), suggesting the accumulation of calcium in mitochondria. Quantification of the mean Calcium Orange AM intensity per MitoTracker Deep Red positive spots confirmed a significant increase in mitochondrial calcium levels (Fig. 3*E*). No increase in Calcium Orange AM intensity was detected in LysoTracker Deep Red–positive spots (*SI Appendix*, Fig. S6), confirming that the increase in intraorganelle calcium was specific to mitochondria.

We next investigated whether specific knockdown of ITPKB was sufficient to increase mitochondria calcium levels in neurons. Consistent with the results from neurons treated with GNF362, neurons transduced with mITPKB shRNA LV exhibited increased Calcium Orange AM fluorescence intensity in MitoTracker Deep Red–positive spots (Fig. 3*F*); no change was observed in the number or size of MitoTracker Deep Red–positive spots (*SI Appendix*, Fig. S6 *A* and *B*) or in the number, size, or calcium concentration of lysosomes (*SI Appendix*, Fig. S6 *D*–*F*). Taken together, these results suggest that ITPKB functions in neurons to negatively regulate the transfer of calcium from ER stores to mitochondria.

### ITPKB Inhibits Calcium-Mediated Mitochondrial ATP Production.

The accumulation of calcium in mitochondria has previously been reported to drive oxidative phosphorylation of adenosine diphosphate via the electron transport chain, thereby increasing adenosine triphosphate (ATP) production (36, 39, 40). We therefore investigated whether ITPKB inhibition increased mitochondrial respiration and ATP production in neurons. To do this, we used the Agilent Seahorse XF Cell Mito Stress Test, which measures four fundamental parameters of mitochondrial function: basal respiration, maximal respiration, spare respiratory capacity, and total ATP production. The neurons pretreated with 10 nM GNF362 for 1 h exhibited an increase in basal respiration, maximal respiration, and ATP production; no change was observed in spare respiratory capacity (Fig. 3 *G*–*I* and *SI Appendix*, Fig. S6 *G*–*J*). We then investigated whether specific knockdown of ITPKB was sufficient to increase mitochondrial respiration. Neurons transduced with mITPKB shRNA LV exhibited increased basal respiration, maximal respiration, and ATP production, with no change in spare respiratory capacity (*SI Appendix*, Fig. S7), consistent with the results following ITPKB inhibition with GNF362 treatment. Neurons transduced with either control or mITPKB shRNA LVs were then cotransduced with AAVs expressing GFP or hITPKB and analyzed by the Seahorse XF Cell Mito Stress Test. In mITPKB shRNA LV-transduced cells, reexpression of hITPKB reduced basal respiration and ATP production (*SI Appendix*, Fig. S7 *D*–*F*). The overexpression of hITPKB in control shRNA LV-transduced cells showed the same direction of effect but was not significantly different from control cells (*SI Appendix*, Fig. S7 *B* and *D*–*F*). Taken together, these data suggest that ITPKB activity and protein level regulates mitochondrial respiration in neurons.

We then confirmed the effect of GNF362 treatment on ATP production by a second method, the Cell Titer Glo luminescence assay, which generates the luminescent product oxyluciferin in the presence of luciferase proportional to the amount of ATP present in a sample (41). Pretreatment with GNF362 for 1 h increased Cell Titer Glo luminescence signal in a dose-dependent manner (Fig. 3*J*), consistent with the results from the Seahorse XF Cell Mito Stress Test. Interestingly, the GNF362-induced increase in ATP production was blocked by pretreatment with either the IP_3_R antagonist 2-APB (2-Aminoethoxy-diphenylborane) or the mitochondrial calcium uniporter (MCU) inhibitors Ruthenium Red and KB-R7943 (*SI Appendix*, Fig. S8 *A*–*H*), suggesting that the effect of GNF362 on mitochondrial respiration requires both calcium efflux from the ER via IP_3_R and calcium import into the inner mitochondrial matrix via the MCU complex. Consistent with this hypothesis, pretreatment with KB-R7943 prevented the increase in respiration induced by GNF362 in the Seahorse XF Cell Mito Stress Test (*SI Appendix*, Fig. S9 *A*–*D*). Inhibition of ER calcium release by the ryanodine receptor (RyR) or Sarco/ER Ca^2+^ ATPase (SERCA) pump had no effect on ATP levels despite increasing cytosolic calcium levels and did not prevent the increase in ATP levels induced by GNF362 treatment (*SI Appendix*, Fig. S8 *I*–*L*).

While transient increases in mitochondrial calcium levels are required for dynamic control of respiratory output under normal physiological conditions, chronic activation of ER-to-mitochondria calcium transfer mechanisms within the cell might lead to overactivation of the mitochondrial respiratory system. Previous reports have shown that chronic elevations in mitochondrial calcium levels lead to an accumulation of reactive oxidative species (ROS) (42). We therefore investigated whether GNF362 treatment increased ROS production. Neurons were treated for 1 h with 10 nM GNF362 and 5 µM CellROX Deep Red reagent, a membrane-permeable dye that is nonfluorescent in its reduced state but becomes brightly fluorescent upon oxidation by ROS, and then imaged live to visualize ROS levels in real time. Neurons treated with 10 nM GNF362 for 1 h exhibited nearly a 50% increase in CellROX Deep Red fluorescence (*SI Appendix*, Fig. S9 *I* and *J*); GNF362 treatment had no effect in cells transduced with mITPKB shRNA LV, suggesting that the effect of GNF362 on ROS accumulation was predominantly driven by ITPKB. Taken together, these results demonstrate that the increase in mitochondrial calcium following ITPKB inhibition is associated with functional changes in mitochondria, including increased respiration, ATP production, and the accumulation of ROS.

### ITPKB Knockdown Inhibits Autophagy Initiation via AMPK and mTOR.

**Fig. 4.**
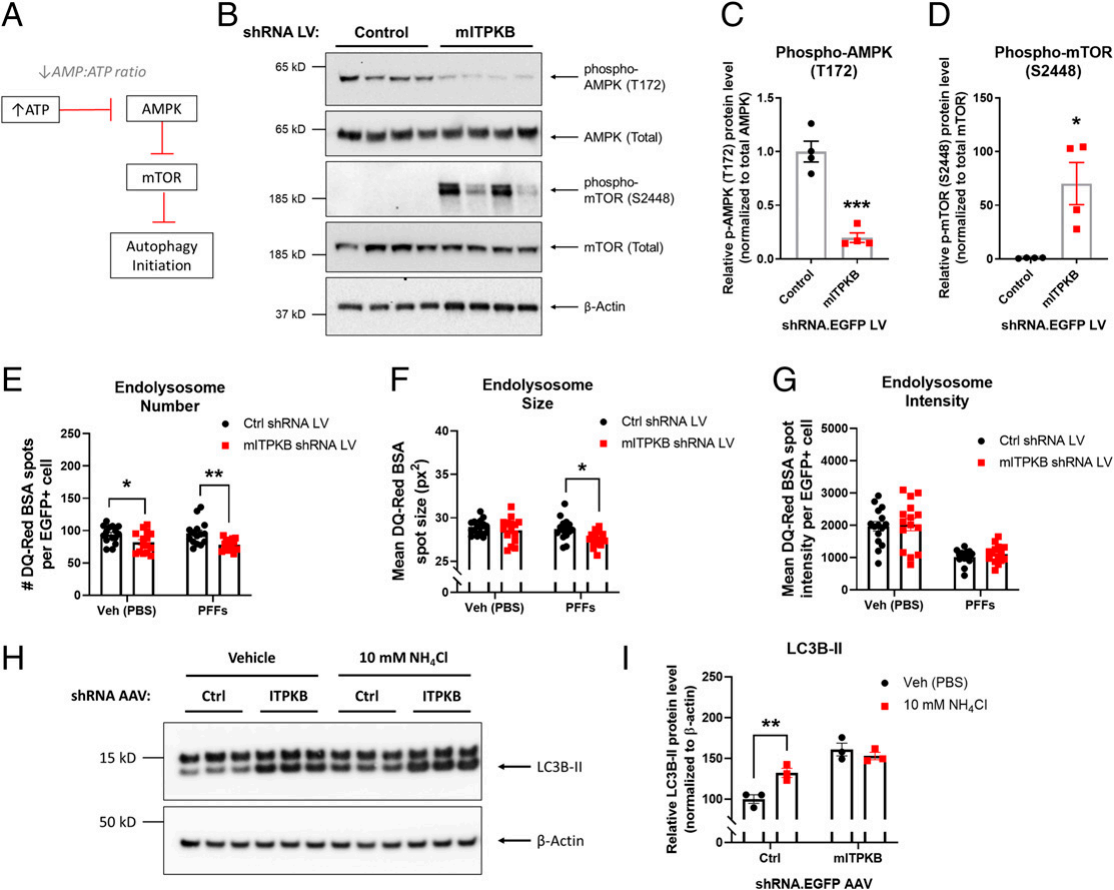
ITPKB knockdown inhibits autophagy initiation and flux via AMPK and mTOR. (*A*) A diagram of AMPK-mediated inhibition of mTOR under conditions of high levels of AMP relative to ATP. Note that a decrease in the AMP:ATP ratio disinhibits mTOR by reducing AMPK activity. (*B*) Representative immunoblots of phosphorylated (T172) and total AMPK, phosphorylated (S2448) and total mTOR, and β-Actin in DIV 20 neurons transduced with control (Ctrl) or mITPKB shRNA LV. (*C*, *D*) Quantification of levels of phospho-T172 AMPK (*C*) and phospho-S2448 mTOR (*D*) in *B*. ***P = 0.0003; *P = 0.0124 by unpaired Student’s *t* test; *n* = 4 wells/group. The bar graphs represent the mean ± SEM. (*E*–*G*) Quantification of the endolysosome number (*E*), size (*F*), and intensity (*G*) in DIV 20 neurons transduced with Ctrl shRNA LV or mITPKB shRNA LV and incubated with 4 µg/mL DQ-Red BSA for 6 h prior to live-cell imaging. The cells were treated with 2 µg/mL PFFs or vehicle (Veh [PBS]) for 11 d. *P < 0.05; **P = 0.0033 by two-way ANOVA with Sidak’s post hoc test; *n* = 15 wells/group. (*H*) A representative immunoblot of LC3B and β-Actin in Ctrl or mITPKB shRNA AAV–transduced neurons (DIV 14) treated with 10 mM NH_4_Cl for 6 h to induce autophagy. (*I*) Quantification of relative LC3B-II levels normalized to Ctrl shRNA AAV + Veh (PBS) group equal to 100 in *H*. **P = 0.0099 by two-way ANOVA with Sidak’s post hoc test; *n* = 3 wells/group. All experiments in this figure were repeated at least twice in independent cultures of primary neurons. The bar graphs represent the mean ± SEM.

Since ITPKB knockdown in neurons increases α-syn pathology, we hypothesized that the increase in mitochondrial ATP production associated with ITPKB inhibition might impact other metabolic processes in the cell, such as autophagy. Other groups have demonstrated that increases in intracellular ATP levels inhibit activation of AMP-activated kinase (AMPK) by reducing the AMP-to-ATP ratio (40). AMPK activity is regulated by phosphorylation at threonine 172 (T172); activation of AMPK in turn negatively regulates the activity of mTOR, a known inhibitor of autophagy initiation (Fig. 4*A*) (43–47). Therefore, we investigated whether ITPKB knockdown impacted AMPK or mTOR activity in neurons. Neurons transduced with mITPKB shRNA LV exhibited reduced levels of pT172 AMPK and increased levels of pS2448 mTOR, as determined by immunoblot analysis (Fig. 4 *B*–*D*). Furthermore, PFF-treated neurons exhibited reduced pT172 AMPK levels compared to control cells (*SI Appendix*, Fig. S10*A*), suggesting that down-regulation of AMPK activity might contribute to pS129 α-syn accumulation in PFF-treated cells. In support of this hypothesis, treatment with the AMPK activator metformin was sufficient to reduce PFF-induced pS129 α-syn pathology in neurons (*SI Appendix*, Fig. S10 *B* and *C*), consistent with previous findings showing protective effects of metformin on pS129 α-syn levels in other PD models (48, 49).

In order to confirm that the increase in mTOR activation following ITPKB knockdown is associated with impaired initiation of autophagy, we used HeLa cells stably overexpressing LC3 dual tagged with mCherry (pH insensitive) and GFP (pH sensitive). Since GFP fluorescence is quenched at acidic pH, this cell line has been widely used to visualize autophagosomes (pH > 6, mCherry+/GFP+ spots) and lysosomes (pH <5, mCherry+ only) in live cells (*SI Appendix*, Fig. S11*A*); LC3 is a microtubule-associated light chain protein required for autophagosome formation and fusion with lysosomes (50, 51). mCherry-GFP-LC3 cells were transfected with siRNAs targeting human ITPKB or a control (nontargeting) sequence and imaged by live-cell fluorescence microscopy 72 h later. Compared to control cells, mCherry-GFP-LC3 cells transfected with hITPKB siRNAs exhibited nearly a 50% reduction in the number of autophagosomes (mCherry+/GFP+ spots) per cell; no change was observed in the number of lysosomes (*SI Appendix*, Fig. S11 *B* and *C*), suggesting that ITPKB knockdown inhibits autophagy at the level of autophagosome formation. An immunoblot analysis of mCherry-GFP-LC3B HeLa cells serum starved for 4 h confirmed that control but not hITPKB siRNA-transfected cells exhibited an increase in phospho-S278 ATG16L1 (*SI Appendix*, Fig. S11 *D* and *E*), a recently described marker of autophagosome initiation (52). This result is consistent with the increase in mTOR activation observed in neurons transduced with mITPKB shRNA LV, which is known to inhibit autophagosome formation in a variety of cell types (43–45).

We next investigated whether the decrease in autophagosome formation following ITPKB knockdown also occurred in neurons. Neurons were transduced with control shRNA LV or mITPKB shRNA LV and incubated with 4 µg/mL DQ-Red bovine serum albumin (BSA) for 6 h, which is internalized by the endolysosomal pathway and labels organelles with acidic pH. Compared to control shRNA LV-transduced cells, neurons transduced with mITPKB shRNA LV exhibited a decrease in the number and size of DQ-Red BSA spots per cell (Fig. 4 *E* and *F*), indicating a decrease in the number of endolysosomes. No difference was observed in mean DQ-Red BSA spot intensity following ITPKB knockdown, although PFF treatment led to an overall reduction in DQ-Red BSA spot intensity (Fig. 4*G*). We next investigated whether the observed decrease in endolysosomes in primary neurons also resulted in a reduction in autophagic flux. DIV 14 neurons were treated with 100 mM NH_4_Cl for 6 h to induce autophagy, as measured by the accumulation of LC3B-II (Fig. 4*H*). Compared to neurons transduced with control shRNA AAV, NH_4_Cl treatment had no effect in neurons transduced with mITPKB shRNA AAV despite higher baseline levels of LC3B-II (Fig. 4 *H* and *I*), suggesting a reduction in autophagic flux. Taken together, these results demonstrate that ITPKB knockdown reduces both autophagosome formation and lysosomal biogenesis in primary neurons, which, in turn, perturbs autophagy flux without impacting lysosomal pH.

### Inhibition of ER-to-Mitochondria Calcium Transfer Reduces α-Syn Pathology.

**Fig. 5.**
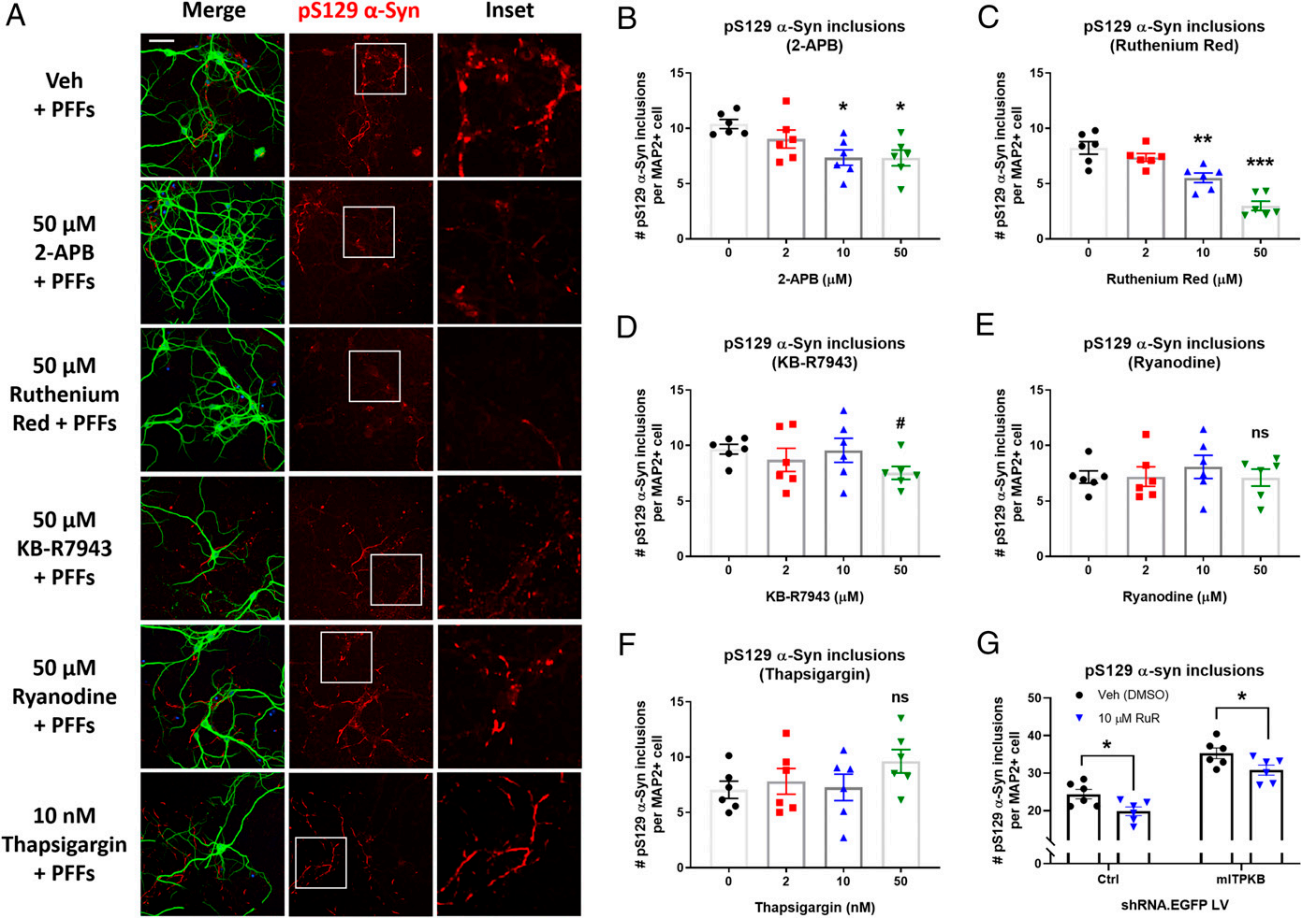
Inhibition of the IP_3_ receptor or mitochondrial calcium uniporter reduces α-syn pathology induced by PFFs. (*A*) Representative images of pS129 α-syn (red), MAP2 (green), and DAPI (blue) immunocytochemistry of neurons pretreated with vehicle (Veh [DMSO]), 2-APB (IP_3_R inhibitor), Ruthenium Red (MCU inhibitor), KB-R7943 (MCU inhibitor), Ryanodine (RyR inhibitor), or Thapsigargin (SERCA inhibitor) for 1 h on DIV 9, followed by cotreatment with 2 µg/mL α-syn PFFs for 8 d. (Scale bar, 20 μm.) (*B*–*F*) Quantification of the number of pS129 α-syn inclusions per MAP2+ cell in *A* for cells treated with 2-APB (*B*), Ruthenium Red (*C*), KB-R7943 (*D*), Ryanodine (*E*), or Thapsigargin (*F*). ^#^*P* = 0.2077; *P < 0.05; **P = 0.0011; ***P = 0.0001 by one-way ANOVA with Dunnett’s post hoc test; *n* = 6 wells/group. (*G*) The number of pS129 α-syn inclusions per MAP2+ cell in Ruthenium Red (RuR)-treated neurons transduced with Ctrl shRNA LV or mITPKB shRNA LV for 11 d. *P < 0.05 by one-way ANOVA with Sidak’s post hoc test; *n* = 6 wells/group. The experiments with RuR were repeated four times in independent primary neuron cultures with different plate layouts. The bar graphs represent the mean ± SEM.

Our results indicate that, in neurons, ITPKB functions to negatively regulate ER calcium release via IP_3_Rs and subsequent import into mitochondria via the MCU. However, whether inhibition of ER-to-mitochondria calcium transfer alone is sufficient to reduce α-syn pathology is unknown. Therefore, we pretreated primary neurons with increasing concentrations of 2-APB (IP_3_R inhibitor), KB-R7943 and Ruthenium Red (MCU inhibitors), Ryanodine (RyR inhibitor), or Thapsigargin (SERCA inhibitor) for 1 h followed by cotreatment with 2 µg/mL mPFFs for 8 d. The cells were fixed on DIV 17 for analysis of pS129 pathology by ICC. As expected, 8-d treatment with PFFs induced a significant accumulation of pS129 α-syn inclusions in neurons (Fig. 5*A*). IP_3_R inhibition by 2-APB treatment resulted in a dose-dependent reduction in the number of pS129 α-syn inclusions per neuron (Fig. 5*B*). Similarly, inhibition of calcium import into mitochondria reduced the number of pS129 α-syn inclusions per neuron (Fig. 5 *C* and *D*). This effect was particularly pronounced in the cells treated with Ruthenium Red, which exhibited a 33% and 64% reduction in the number of inclusions per neuron following treatment with 10 µM and 50 µM Ruthenium Red, respectively (Fig. 5*C*). KB-R7943 treatment had a more modest effect on pS129 α-syn pathology compared to Ruthenium Red, perhaps due to the known off-target activity of KB-R7943 on additional ion channels (53). In contrast, treatment with Ryanodine or Thapsigargin had no effect on the accumulation of pS129 α-syn pathology (Fig. 5 *E* and *F*), suggesting that ER-to-mitochondria calcium release specifically via the IP_3_R, and not other ER calcium channels, is able to regulate α-syn aggregation in neurons. Furthermore, Ruthenium Red treatment decreased pS129 α-syn inclusions to a similar extent in both control shRNA– and mITPKB shRNA LV-transduced neurons (Fig. 5*G*), confirming that ITPKB acts upstream of calcium entry into the inner mitochondrial matrix via MCU.

### The Protective ITPKB Allele Is Associated with an In-Frame Protein Coding Deletion that Increases Localization to the ER.

We next sought to investigate how the genome-wide significant variant in the ITPKB gene locus (rs4653767-C) might impact protein function to protect against PD risk. Since our prior experiments in neurons demonstrated that increasing ITPKB protein levels could protect against the accumulation of neuropathology (Figs. 1 and 2), we hypothesized that rs4653767-C was associated with increased expression of ITPKB. A meta-analysis of expression quantitative trait loci (eQTL) in cortex tissue at SNP rs4653767-C compared to the T allele indicated that the protective variant was not associated with increased expression across more than 10 independent cohorts of human cortex samples (MetaBrain eQTL, *SI Appendix*, Fig. S12*A* and Table S1). We also did not detect a significant change in ITPKB transcript or protein expression in PD amygdala samples compared to nondemented control tissues (*SI Appendix*, Fig. S13 *A*–*E*). We chose to analyze the amygdala because it is a brain area severely affected by α-syn pathology in patients with Braak stages IV to VI without the presence of overt neurodegeneration (54), allowing us to exclude the possibility that any observed changes are due to changes in cell type composition alone. These results suggest that the reduced risk of PD associated with the rs4653767-C allele is not explained by changes in ITPKB expression.

Because we expect the lead GWAS SNP to be either the disease variant or in high linkage disequilibrium with the disease variant, we decided to investigate whether rs4653767-C was associated with any other known functional variants in the ITPKB gene locus. The rs147889095 allele encodes a nine-nucleotide deletion in the protein-coding sequence of the ITPKB gene, which produces an in-frame three-amino-acid deletion (Gly94-Ser95-Ser96, or “ΔGSS”) in the ITPKB protein-coding sequence (55). Chi-square analysis of a cohort of 1,006 individuals of European ancestry (non-PD) using the LD pair tool available via the NIH revealed that the protective rs4653767-C allele is in linkage disequilibrium with the rs147889095 deletion allele (*SI Appendix*, Fig. S12*B*; *R*^2^ = 0.9954, Chi-square = 1,001.3652, *P* < 0.0001). Of the 691 patients with the wild-type ITPKB allele (rs4653767-T), 690 possess the full-length (FL) protein-coding sequence of rs147889095 (99.86%), while only 1 patient (0.14%) had the ΔGSS allele (*SI Appendix*, Fig. S12 *C* and *D*). In contrast, all 315 patients (100%) with the protective rs4653767-C allele also possessed the ΔGSS allele of rs147889095. This result raises the possibility that the ΔGSS variant in the protein-coding sequence of ITPKB might be responsible for the reduced PD risk associated with the rs4653767-C allele.

To determine the effects of the ΔGSS deletion on ITPKB function, we engineered an AAV construct expressing ΔGSS hITPKB-Flag under the same promoter as our FL hITPKB-Flag construct and packaged both plasmids into AAVs. We first investigated whether ΔGSS impacted the ability of FL hITPKB-Flag to reduce pS129 α-syn pathology in PFF-treated neurons. Similar to FL hITPKB-Flag, overexpression of ΔGSS hITPKB-Flag reduced levels of insoluble pS129 α-syn pathology (*SI Appendix*, Fig. S12 *E*–*G*). There was no difference in the levels of pathology in neurons transduced with ΔGSS compared to FL hITPKB AAV even after controlling for hITPKB-Flag overexpression levels (*SI Appendix*, Fig. S12 *H* and *I*). There was also no significant change in pT172 AMPK or pS2448 mTOR levels in ΔGSS compared to FL hITPKB AAV–transduced neurons, although both increased pT172 AMPK and decreased pS2448 mTOR levels, respectively (*SI Appendix*, Fig. S12 *J*–*L*), as expected from our prior analysis of AMPK and mTOR pathway activation in FL hITPKB AAV–transduced neurons (Fig. 4 *A*–*D*).

We next investigated the protein domains affected by the ΔGSS deletion in ITPKB. The ΔGSS (Gly94-Ser95-Ser96) deletion occurs in the N terminus of hITPKB proximal to the F-Actin targeting domain (amino acids 107 to 180). A prior study reported that the deletion of the entire N terminus of hITPKB (amino acids 1 to 262) disrupted targeting of hITPKB to the F-Actin cytoskeleton and resulted in a complete relocalization of hITPKB near the ER (56). Since our results indicate that ITPKB mediates ER-to-mitochondria calcium exchange, we investigated whether the ΔGSS deletion might impact intracellular targeting of ITPKB in neurons. The neurons were transduced with either FL or ΔGSS hITPKB-Flag AAV on DIV 5 and fixed for ICC analysis on DIV 18. Anti-Flag immunostaining indicated that ΔGSS hITPKB-Flag exhibited an 11% increase in colocalization with calreticulin (ER marker)-positive immunofluorescence compared to FL hITPKB-Flag (*SI Appendix*, Fig. S12 *M* and *N*), suggesting that the ΔGSS deletion impairs ITPKB interaction with F-Actin, leading to the accumulation of ITPKB at the ER. No difference in expression was observed for FL compared to ΔGSS hITPKB-Flag (*SI Appendix*, Fig. S12*O*), confirming that the observed difference in localization could not be attributed to differences in FL versus ΔGSS expression level. These results suggest that the protection associated with rs4653767-C may be mediated by a second protein-coding allele (rs147889095) in high linkage disequilibrium with rs4653767-C, which produces a protein variant predisposed to localizing to the subcellular site of action required for its impact on α-syn pathology. Consistent with this observation, neurons overexpressing ΔGSS hITPKB-Flag exhibited increased neurite length compared to control cells as early as 7 d posttransduction (*SI Appendix*, Fig. S14 *A* and *B*); FL ITPKB-Flag AAV transduction also increased neurite length, although the difference was not statistically significant prior to 10 d posttransduction (*SI Appendix*, Fig. S14*C*). These results suggest that in addition to reducing α-syn pathology, ΔGSS ITPKB also exerts a subtly greater impact on neuronal health relative to FL hITPKB. The mild effect of the ΔGSS allele is consistent with the relatively small OR (OR = 0.92) and common allele frequency for the protective GWAS ITPKB allele associated with reduced sporadic PD risk. However, the full effect of the ΔGSS deletion on endogenous ITPKB function, as well as whether the deletion is sufficient to protect against PD risk, is unknown.

### ITPKB Overexpression Is Associated with Reduced α-Syn Pathology In Vivo.

We next decided to investigate whether either FL or ΔGSS overexpression reduces α-syn pathology in vivo. Postnatal day 0 C57BL/6J mice were injected intracerebroventricularly with 1E11 genome copies of GFP control, FL hITPKB-Flag, or ΔGSS hITPKB-Flag AAVs and aged to 2 mo prior to unilateral injection of 10 µg mPFFs or saline (PBS) directly into the striatum. Ipsilateral brain tissues were dissected 30 d postinjection and analyzed by biochemical fractionation. An immunoblot analysis of the soluble lysates confirmed robust expression of the GFP control and hITPKB-Flag (FL and ΔGSS) AAVs (*SI Appendix*, Fig. S15 *A* and *B*). An immunohistochemistry analysis of the contralateral cortex confirmed neuronal expression of GFP control, FL hITPKB-Flag, and ΔGSS hITPKB-Flag AAVs (*SI Appendix*, Fig. S15*C*). As expected, immunoblot analysis of the 2% SDS fractions confirmed an accumulation of insoluble, pS129 α-syn aggregates in ipsilateral cortex tissues of PFF- compared to PBS-injected mice (*SI Appendix*, Fig. S16*A*). Mice injected with ΔGSS hITPKB-Flag AAV exhibited a strong trend toward reduced insoluble pS129 α-syn levels compared to control mice injected with GFP AAV (*SI Appendix*, Fig. S16 *B*–*D*).

We next evaluated the contralateral cortex for markers of neuroinflammation. The sections were stained for immunoreactivity to GFAP and Iba1 which label astrocytes and microglia, respectively. PFF injection alone did not induce an increase in astrocytic or microglial reactivity, as expected (*SI Appendix*, Fig. S16 *E*–*G*). Furthermore, no differences in the number of GFAP-positive cells per 100 µm^2^ cortex tissue was observed between any of our four treatment groups (*SI Appendix*, Fig. S16 *E* and *F*). In contrast, the number of Iba1-positive microglia was increased in the ΔGSS hITPKB AAV compared to the FL hITPKB AAV group (*SI Appendix*, Fig. S16*G*), suggesting that the ΔGSS deletion in hITPKB might impact microglial function via noncell autonomous signaling. Interestingly, the number of Iba1-positive cells in the contralateral cortex was strongly correlated with reduced pS129 α-syn pathology in the same tissues (*SI Appendix*, Fig. S16*H*), raising the possibility that the ΔGSS deletion in ITPKB in neurons may alter the processing of α-syn aggregates by nonneuronal cells such as microglia. Taken together, these results provide support that the protective effects of ITPKB overexpression observed in primary neurons are biologically relevant in vivo and highlight the need for further investigation into the role of ITPKB in both neuronal and glial biology as well as PD pathogenesis.

### MCU Regulatory Subunits Are Dysregulated in Human PD.

**Fig. 6.**
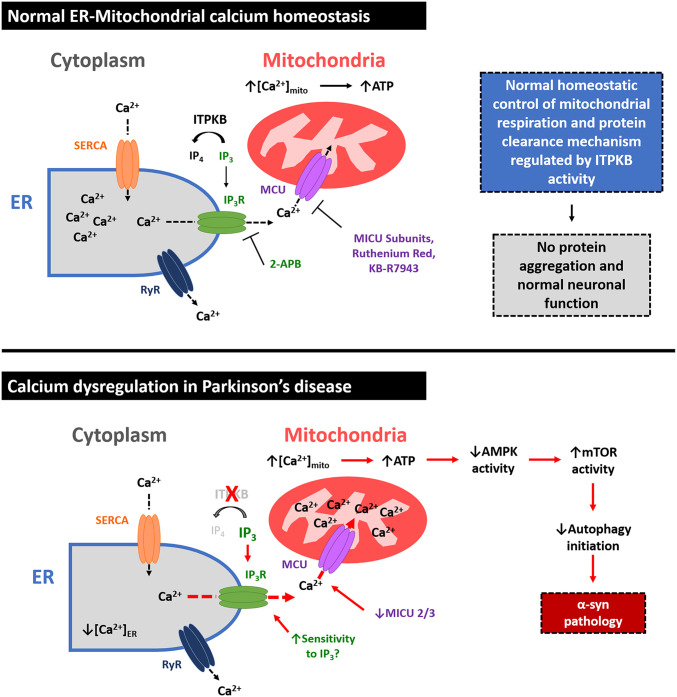
Cellular model for ITPKB function in PD. In healthy neurons (*Top*), the ER is the predominant storage organelle for intracellular calcium (Ca^2+^), which is transiently released in response to various upstream signaling events to mediate diverse cellular functions. ER Ca^2+^ can be released through either the IP_3_R or the Ryanodine receptor (RyR), while the ER-cytosol Ca^2+^ gradient is preserved by SERCA. In response to increases in intracellular IP_3_ levels, Ca^2+^ is released from the ER via IP_3_Rs, leading to Ca^2+^ accumulation in mitochondria mediated by transport into the inner mitochondrial matrix via the mitochondrial calcium uniporter (MCU). Transient increases in mitochondrial calcium levels ([Ca^2+^]_mito_) drive the production of ATP from ADP by oxidative phosphorylation. ITPKB enzymatic activity negatively regulates IP_3_-mediated ER calcium release by converting IP_3_ to IP_4_, thereby protecting cells from overproduction of ATP and accumulation of ROS. The MCU subunits MICU1, MICU2, and MICU3 serve as Ca^2+^ sensors to control homeostatic levels of Ca^2+^ import into mitochondria. In PD (*Bottom*), loss of ITPKB activity, changes in MCU regulatory subunit composition, and/or chronic increases in intracellular IP_3_ levels or sensitivity of IP_3_Rs to IP_3_ may contribute to increased [Ca^2+^]_mito_, leading to ROS generation, mTOR activity, and the accumulation of misfolded proteins, including α-syn aggregates. Pharmacological agents that positively modulate ITPKB activity or inhibit ER-to-mitochondria calcium exchange may represent a new therapeutic approach for the treatment of synucleinopathies such as PD.

Our findings regarding ITPKB function suggest that chronic ER-to-mitochondria calcium release via IP_3_Rs leads to PD-like neuropathology in primary neurons. However, whether mitochondrial calcium handling is dysregulated in the brains of PD patients is unknown. We therefore investigated whether genes in the IP_3_/mitochondrial calcium pathway, specifically the regulatory subunits of the MCU complex (MICU1, MICU2, and MICU3), might be dysregulated in human PD tissues at the protein level. MICU1, MICU2, and MICU3 are the primary gatekeepers for MCU-mediated transport of calcium into mitochondria and function to inhibit calcium import at resting cytosolic concentrations (57, 58). We analyzed amygdala tissues from patients with Braak stages IV to VI PD with confirmed pathology for protein expression levels of MICU1, MICU2, and MICU3. While MICU1 protein levels were unchanged, MICU2 and MICU3 were down-regulated by an average of 66% and 68%, respectively (*SI Appendix*, Fig. S17 *A*–*D* and Table S2). Combined with our prior findings in primary neurons, these results suggest a biological mechanism for the etiology of Lewy body pathology in sporadic PD patients without SNCA mutations (Fig. 6), whereby select neuronal populations are predisposed to the accumulation of α-syn aggregates due to the sensitization of their mitochondria to calcium overload.

## Discussion

While the symptoms and neuropathology of PD have been known for decades, the underlying causes of sporadic disease remain poorly understood. Here, we used a combination of primary neuron, human iPSC, and animal models to demonstrate that ITPKB, a candidate PD-associated gene, regulates PD-like neuropathology. We show that knockdown or inhibition of ITPKB increases the accumulation of pS129 α-syn aggregates in PFF-treated neurons, which was associated with increased calcium release from the ER, elevated mitochondrial calcium levels, and enhanced mitochondrial respiration. This increase in respiration resulted in increased ATP production, reduced AMPK activity, increased mTOR activity, and disrupted initiation of autophagy, leading to reduced flux. Our results suggest that the increase in mitochondrial calcium was particularly relevant for α-syn aggregation, as pharmacological inhibition of either calcium release from IP_3_Rs or calcium import into the inner mitochondrial matrix via the MCU was sufficient to reduce the number of pS129 α-syn aggregates in PFF-treated neurons. Conversely, overexpression of ITPKB reduced mitochondrial respiration, mTOR activation, and pS129 α-syn aggregation. Taken together, our results position ITPKB as a negative regulator of several cellular processes known to contribute to α-syn aggregation and toxicity in PD, including mitochondrial/ER stress and impaired autophagic clearance of intracellular aggregates.

The ability of MCU inhibitors to reduce α-syn pathology in PFF-treated neurons is particularly notable. The MCU complex is composed of transmembrane domains which form the channel pore and regulatory subunits (MICU1, MICU2, and MICU3) that bind calcium in the intermembrane space (57, 59, 60). MICU1 and MICU2 are ubiquitously expressed (57, 58), while MICU3 is a brain-specific MICU isoform (61). All three MICU subunits have low binding affinities for calcium, allowing the MCU complex to permit calcium entry only at high concentrations and in response to the conformational change in the channel pore caused by MICU binding to calcium ions. While the relative influence of MICU1, MICU2, and MICU3 on MCU function remains highly controversial (62, 63), it is generally accepted that MICU subunits prevent excessive transport of calcium into mitochondria at physiological calcium levels. Our analysis of PD amygdala tissue revealed that levels of MICU2 and MICU3 are reduced in the PD brain (*SI Appendix*, Fig. S17), raising the possibility that increased mitochondrial calcium entry caused by dysregulation of MCU regulatory subunit composition is a novel feature of PD. This possibility is supported by the recent GWAS that identified common SNPs in the gene locus of MICU3 as candidate risk alleles for sporadic PD (19, 20). Furthermore, primary neurons expressing LRRK2 mutations exhibit increased mitochondrial calcium uptake (64), while MCU inhibition protects against dopaminergic neuron loss in PINK1 mutant zebrafish (65) and rescues dendritic shortening induced by LRRK2 mutations in primary neurons (64). Our data showing down-regulation of MICU2 and MICU3 in human PD brain combined with the observation that MCU inhibition reduces α-syn pathology in primary neurons corroborates the potential role for dysfunction of the MCU complex in PD pathogenesis.

Our results also highlight ER calcium release via the IP_3_R as a mediator of α-syn pathology in neurons. The IP_3_R has been previously implicated in several neurodegenerative movement disorders. Missense mutations in the gene encoding the IP_3_R (ITPR1) cause Spinocerebellar Ataxia type 15 (66), while the polyglutamine repeat expansion proteins ataxin-2 and huntingtin bind to the IP_3_R C terminus and increase its sensitivity to IP_3_ (67, 68). IP_3_R inhibition also protects against glutamate-induced apoptosis in primary medium spiny neurons cultured from mutant HTT transgenic mice (68). While the involvement of the IP_3_R in PD is at this time unknown, elevated IP_3_ levels in the 6-OHDA rat model of PD have been reported (69). The results from our study combined with the published literature suggest that IP_3_-mediated calcium signaling may be a novel pathway that contributes to mitochondrial dysfunction or disease progression in PD.

IP_3_R and the mitochondrial calcium uptake machinery function cooperatively to preserve the structural integrity of MAMs, a critical intracellular signaling hub in neurons and glia. IP_3_Rs are concentrated at MAMs and mediate calcium transfer between the ER and intracellular organelles, including mitochondria and lysosomes. α-Syn has previously been shown to localize to MAMs, where α-syn regulates mitochondrial fission and fusion (70). α-Syn oligomers directly interact with mitochondrial membranes and sensitize mitochondria to calcium-induced dysfunction and cytochrome *c* release in an MCU-dependent manner (71, 72). MAMs are also known to regulate a variety of cellular processes linked to PD, including lipid metabolism, mitochondrial morphology, ROS-induced stress, autophagy, and apoptosis (73), and are the site of formation for both autophagosomes (74) and the inflammasome (75). Our findings suggest that dysfunction of ER–mitochondrial calcium signaling at MAMs may contribute to the development of PD due to either enhanced IP_3_R activity, compromised ITPKB or MCU function, or the sensitization of mitochondria to calcium-induced stress by α-syn aggregates.

Our results also identify AMPK as a downstream mediator of the IP_3_ signaling pathway that functions as a metabolic sensor for upstream calcium dyshomeostasis in neurons. We show that AMPK activity negatively correlates with both mTOR activity and α-syn pathology in primary neurons, suggesting AMPK activation as a potential therapeutic strategy in PD capable of normalizing cellular metabolism, reducing neuropathology, or both. In support of this hypothesis, the AMPK activator metformin, which is an approved therapy for diabetes mellitus, has been shown to be protective in multiple cellular and animal models of PD (48, 49, 76, 77), an observation that we now extend to the neuronal PFF α-syn seeding model. These findings may be therapeutically relevant due to the increased incidence of PD in patients with diabetes (78).

Multiple GWAS have validated the rs4653767-C allele in the gene locus of ITPKB as a protective SNP against the development of sporadic PD. However, exactly how the rs4653767-C allele impacts ITPKB expression or function is unknown. An eQTL meta-analysis of rs4653767-C indicated no change in the transcript level compared to rs4653767-T in whole cortex tissue, suggesting that the protection against PD is not mediated by increased expression alone. However, we cannot rule out that rs4653767-C exerts brain region– or cell type–specific effects on ITPKB expression, and it is also possible that changes in expression or regulation of ITPKB at the protein level play a role in the protection associated with rs4635767-C. In light of this possibility, it is noteworthy that the rs4653767-C allele is in high linkage disequilibrium with a second allele, rs147889095, which encodes an in-frame protein coding deletion (ΔGSS) in ITPKB. Our current data demonstrate that ΔGSS hITPKB is equally effective at reducing pS129 α-syn pathology in primary neurons, suggesting that the neuroprotection associated with rs4653767-C might not be mediated solely by its effects on α-syn pathology. In primary neurons, ΔGSS hITPKB exhibited increased localization to the ER compared to FL hITPKB, suggesting that subtle changes in the interaction of ITPKB with MAMs may have synergistic effects on ER–mitochondria calcium homeostasis and neuronal health in the aging brain. In the context of the experiments performed here, it is also possible that any subtle effects of ΔGSS on ITPKB function that would be otherwise observable at endogenous expression levels are masked by the overexpression levels attained by chronic AAV transduction. Taken together, our results highlight the need for further research into the function of ΔGSS and FL ITPKB in order to elucidate the potential neuroprotective mechanisms that are tractable for therapeutic modulation in PD.

In summary, our findings demonstrate that ITPKB activity and protein levels regulate α-syn pathology in cellular and animal models of PD. We propose a model of ITPKB function in PD whereby ITPKB activity serves as a negative regulator of α-syn aggregation by inhibiting ER-to-mitochondria calcium release (Fig. 6). Pharmacological agents that positively modulate ITPKB activity or inhibit ER-to-mitochondria calcium exchange may represent a new therapeutic approach for the treatment of synucleinopathies such as PD.

## Materials and Methods

Primary neuron experiments were performed by isolating embryonic day 17 cortex tissue from C57BL/6J mice according to protocols approved by the Biogen Institutional Animal Care and Use Committee. Cells were cultured in Poly-d-lysine coated cellware for up to 20 d in vitro prior to analysis by live-cell imaging, immunoblot, immunocytochemistry, and Seahorse assays for mitochondrial function. Please refer to the *SI Appendix* for a complete and detailed description of all experimental materials and methods.

## Supplementary Material

Supplementary File

## Data Availability

All study data are included in the article text and supporting information.
