# Supplementary material for: The Parkinson’s disease-associated gene ITPKB protects against α-synuclein aggregation by regulating ER-to-mitochondria calcium release
Source: Proc Natl Acad Sci U S A. 2020 Dec 21;118(1):e2006476118. doi: 10.1073/pnas.2006476118 (PMC7817155; doi:10.1073/pnas.2006476118)
Supplement: Supplementary File [file pnas.2006476118.sapp.pdf]

# Apicco et al., The Parkinson's disease associated gene ITPKB protects against α-synuclein aggregation by regulating ER-to-mitochondria calcium release.

## Supporting Information Appendix – Materials and Methods (pertaining to Figures 1 to 5)

### Primary Neuron Culture

Mouse cortical neurons were isolated and homogenized from timed-pregnant C57BL/6J mouse embryos (Charles River) on embryonic day 17 (E17) according to Biogen Institutional Animal Care and Use Committee (IACUC) guidelines. Briefly, cortex tissues were dissected on ice in Hank's Buffered Saline Solution (HBSS) without calcium or magnesium (Gibco, Cat# 21-022-CV), carefully separated from the meninges, washed 3x in ice-cold HBSS, and then incubated at 37°C with 0.25% Trypsin-EDTA and 1 mg/ml DNase I (Sigma, Cat# DN25-10MG) for 15 min. Cell were resuspended in Neurobasal media (Gibco, Cat# 21103) containing 10% fetal bovine serum (FBS) and 1x GlutaMAX (Gibco, Cat# 35050) and filtered through a 100 µm cell strainer. For all imaging experiments and plate-based assays, neurons were plated at a density of 20,000 cells per well in Corning Biocoat Poly-D-Lysine 96-well plate Cellware (ThermoFisher Scientific, Cat#356640) with black wells and clear bottom with lid. For biochemistry experiments, neurons were plated at a density of 500,000 cells per well in Corning Biocoat Poly-D-Lysine 12-well plate Cellware (ThermoFisher Scientific, Cat# 087-74-269). 1.5 h later, Plating media was aspirated and replaced with Neurobasal Feeding media supplemented with 2% B27 supplement (Gibco, Cat# 17504-044), 1x GlutaMAX, and 1x Pen/Strep (Gibco, Cat# 15140122). Unless specified otherwise in this manuscript, media was changed via 50% fresh Feeding media addition on days in vitro (DIV) 5 and 9. For experiments using lentiviruses (LVs) or adeno associated viruses (AAVs), virus was added with fresh media on DIV 5. Except for preformed fibril (PFF) experiments, all cells were imaged, fixed, or lysed for subsequent analysis on DIV 14 or 15. For PFF experiments, PFFs were added on DIV 9 with fresh media.

### shRNA and Overexpression Plasmids

shRNA sequences were cloned into a mammalian pLL3.7 entry vector under the H1 promoter (Addgene). The shRNA sequence targeting murine ITPKB (mITPKB shRNA) was ACCGCCTGAGAGTTCCATAGTTTGTTCAAGAGACAAACTATGGAACTCTCAGGCTTTTTTGGAAA; an shRNA sequence targeting the firefly luciferase enzyme (Control shRNA) was used as a negative control (TGGATTCCAATTCAGCGGGAGCCACCACCTGATGAAGCTTGATCGGGTGGCTCTCGCTGAGTTGGA ATCCTTTTTTC). Lentivirus was produced using the ViraPower HiPerform Lentiviral Expression Kit, according to manufacturer's instruction (Invitrogen, Cat# K533000). For overexpression plasmids, the full length (FL) human ITPKB (hITPKB) gene sequence or the ΔGSS deletion gene were ordered from Origene and cloned into pLenti6 expression vectors with a C-terminus Myc-Flag tag. Plasmids were then shipped to Packgene Biotech (Worcester, MA) for cloning into AAV entry vectors under the CAG promoter followed by packaging into AAV using the PHP.B serotype. PHP.B serotype was chosen based on enhanced transduction efficiency relative to AAV9 previously reported in C57BL/6J mice.

### Biochemical Fractionation

For *in vitro* experiments, cells were lysed in ice-cold 110 µl 1x Tris-buffered saline (TBS, 25 mM Tris, 0.15M NaCl; ThermoFisher Scientific, Cat# 28358) supplemented with 1% Triton X-100 and Halt protease/phosphatase inhibitor cocktail with EDTA (Pierce, Cat# 78442). Cells were lifted using cell scrapers, and the lysates were transferred to 1.5 ml microcentrifuge tubes followed by centrifugation at 20,000g for 45 min at 4°C. Supernatants (1% Triton X-100 soluble fractions) were aliquoted into new microcentrifuge tubes and stored at -80°C until day of analysis. Cell pellets were resuspended in 110 µl 1% Triton X-100 lysis buffer, vortexed, and centrifuged a second time at 20,000g for 45 min at 4°C to wash the pellets. After the second spin, the supernatants were removed and discarded. 40 µl lysis buffer supplemented with 2% SDS was added to the cell pellets, which were then brought to room temperature (RT). The cell pellet/SDS buffer mixtures were then vortexed and sonicated using a Q Sonica water bath sonicator (30 seconds on, 20 seconds off for 2 minutes at Amplitude 50). Following sonication, cells were centrifuged at 20,000g for another 45 min at RT. Supernatants (SDS fractions) were then aliquoted into new microcentrifuge tubes and stored at -80°C. Protein concentrations were determined by bicinchoninic acid (BCA) protein quantitation assay (Pierce, Cat# 23225). For autophagy flux analysis, cells treated with

or without 10 mM $NH_4Cl$ (Sigma, Cat# A9434) for 6 h were collected in radioimmunoprecipitation assay (RIPA) buffer (Boston BioProducts, Cat# BP-115) supplemented with Halt protease/phosphatase inhibitor cocktail with EDTA. Samples were prepared for Western blot analysis using Bolt 4X Lithium Dodecyl Sulfate (LDS) sample buffer (Invitrogen, Cat# B0007) and 10X Bolt sample reducing agent (Invitrogen, Cat# B0004) prior to gel electrophoresis by Bolt 4-12% Bis-Tris SDS-PAGE (polyacrylamide gel electrophoresis) gels (Invitrogen, Cat# NW04125BOX). Unless specified otherwise, 10 µg and 3 µg total lysates were loaded per well for soluble and insoluble samples, respectively. Western blotting was performed as described below.

### Western blotting

Samples were separated by SDS-PAGE and transferred to polyvinylidene difluoride (PVDF) or nitrocellulose membranes using the iBlot 2 Gel transfer system (ThermoFisher Scientific, Cat# IB21001) according to manufacturer's instructions. Membranes were washed 1x in TBS/0.05% Tween-20 (TBS-T) followed by blocking in 5% nonfat dry milk (NFDM)/TBS-T for 1 h. For immunoblotting using the antibody to α-synuclein aggregates phosphorylated at serine 129 [EP1536Y] (abcam, Cat# ab51253), membranes were incubated in 0.4% paraformaldehyde (PFA)/PBS for 15 min prior to blocking in order to induce protein crosslinking to the membrane. After blocking, membranes were rinsed 3x in TBS-T, 3 min each and incubated at 4°C overnight in primary antibodies diluted in 2% bovine serum albumin (BSA)/TBS-T (see below for detail regarding antibodies and dilutions used for Western blotting). The next day, membranes were washed 3x in TBS-T, 10 min each followed by incubation in horseradish peroxidase (HRP)-conjugated goat anti-mouse IgG or anti-rabbit IgG secondary antibodies (Jackson ImmunoResearch) diluted 1:10,000 in 1% NFDM/TBS-T. After 2 h incubation in secondary antibodies, membranes were washed 3x in TBS-T, 10 min each. Immunoblot detection was performed using Amersham ECL Prime Western Blot Detection Reagent (GE Healthcare, Cat# RPN2232) or SuperSignal West Femto Maximum Sensitivity Substrate (ThermoFisher Scientific, Cat# 34095) followed by imaging using a Bio-Rad ChemiDoc Imager. Chemiluminescent densitometry analysis of Western blot images was performed using Bio-Rad Image Lab software.

### Immunocytochemistry

For PFF experiments, MCNs were rinsed in 1x PBS and fixed in 4% PFA/PBS for 15 min at RT. Fixed cells were washed 3x in PBS, 3 min each followed by immunocytochemistry (ICC) or storage at 4°C in 0.01% sodium azide/PBS until day of ICC. Fixed cells were permeabilized in 0.1% Triton X-100/PBS for 20 min followed by blocking in 0.05% Triton X-100/PBS (PBS-T) supplemented with 5% normal goat serum (NGS) for 1 h. Cells were then incubated overnight with primary antibodies diluted in 2% BSA/PBS-T at 4°C, as described below. The next day, cells were washed 3x in PBS-T, 15 min each followed by incubation with goat anti-mouse IgG, anti-rabbit IgG, and/or anti-Chicken IgY secondary antibodies conjugated with Alexa Fluor fluorescent dyes (Invitrogen, Cat# A32728, A-11036, A32933) for 2 h at RT protected from light by aluminum foil. All secondary antibodies were diluted 1:700 in 5% NGS/PBS-T block buffer supplemented with 0.5 µg/ml DAPI (Invitrogen, Cat# 62248). After secondary antibody labeling, cells were washed 3x, 15 min each in PBS-T and stored in 0.01% sodium azide/PBS at 4°C until imaging and analysis by confocal microscopy.

### Live-Cell & Confocal Imaging

For live-cell imaging experiments, cells were pre-incubated with the following cell permeant fluorescent dyes (added to conditioned cell media): NucBlue Hoechst 33342 (Invitrogen, Cat# R37605, 1:200 dilution), Calcium Orange AM (Invitrogen, Cat# C3015, Working Concentration = 2 µM), MitoTracker Deep Red FM (Invitrogen, Cat# M22426, Working Concentration = 5 nM), LysoTracker Deep Red (Invitrogen, Cat# L12492, Working Concentration = 5 nM), DQ-Red BSA (Invitrogen, Cat# D12051, Working Concentration = 4 µg/ml), and CellROX Deep Red (Invitrogen, Cat# C10422, Working Concentration = 5 µM). After 1 h, cells were gently rinsed 3x with pre-warmed Dulbecco's PBS (DPBS) and immediately imaged at 40x magnification at 37°C using the Perkin Elmer Opera Phenix high-content confocal imager. After imaging, cells were then lysed in 1x Passive Lysis buffer (Promega, Cat# E1941) to be compatible with subsequent analysis by Cell Titer Glo (Promega, Cat# G7571) luminescence assays, which were performed according to manufacturer's instructions.

### Image Analysis

Live-cell and fixed-cell ICC experiments were imaged using the Perkin Elmer Opera Phenix high-content confocal imager. Images were captured using Harmony software and transferred to the Columbus image

analysis platform for subsequent analysis by batch processing. All statistical analysis was performed using Graphpad Prism software. Whenever possible, the figure legends in this manuscript provide the exact p values, sample size, statistical test and post-hoc correction used, if applicable, and the number of biological replicate experiments performed; individual data points and/or box-and whisker plots are shown for all figures.

### Agilent Seahorse Mito Stress Test Assay

For Seahorse experiments, primary neurons were isolated as described above, and plated at a density of 20,000 cells per well in 96-well XF Cell Culture Microplates pre-coated with 100 µg/ml Poly-D-Lysine (Millipore, Cat# A-003-E) and 50 µg/ml Laminin (Invitrogen, Cat# 23017-015) overnight. Mitochondrial oxygen consumption was measured according to the instructions of the XF Cell Mito Stress Test Kit (Agilent Technologies, Cat# 103015-100). On the day of the assay (DIV14-DIV16), cells were washed 2x with and incubated in Assay Medium (10mM pyruvate, 10mM glucose in Agilent Seahorse XF DMEM Medium pH 7.4, Cat# 103575-100) in a 37°C non-$CO_2$ incubator for 1 h. Oxygen consumption rates (OCRs) were measured with an XF96 extracellular flux analyzer (Agilent Seahorse) at baseline, after addition of 1 µM oligomycin to evaluate respiration associated with cellular ATP production, after addition of 1.5 µM Carbonyl cyanide-4 (trifluoromethoxy) phenylhydrazone (FCCP) to evaluate uncoupled respiration, and after addition of 0.5 µM rotenone/antimycin A to measure non-mitochondrial respiration. Upon completion of analysis, cells were fixed in 2% paraformaldehyde for 20 min at room temperature, washed 2x with PBS and nuclei were stained using NucBlue (Invitrogen, Cat# R37606). The number of cells per well were imaged using the Permin Elmer Opera Phenix high-content automated imager, as described above (Opera Phenix, Perkin Elmer). Raw OCR values were then normalized to the number of cells per well.

## Supporting Information Appendix – Materials and Methods (pertaining to Figures S1 to S17)

### Generation of human iPSC derived neurons

Human iPSCs were obtained from a patient with a SNCA gene triplication, as described previously (Zambon et al. 2019, PMID: 30753527). iPSCs were thawed in mTESR Plus basal media (StemCell Technologies, Cat# 05826) supplemented with 1x mTESR Plus Supplement (StemCell Technologies, Cat# 05827) plus 10 µM Rock inhibitor Y-27632 (Sigma, Cat# Y0503), and seeded in 6 well plates pre-coated with hESC-qualified Matrigel (Corning, Cat# 354277). Cells were maintained in mTESR Complete media. When iPSCs reached ~75% confluence, cells were passaged 1:10 into a new Matrigel-coated 6 well plate. On the next day (Day 1), cells were fed by complete media exchange with fresh mTESR Complete media. On Day 2, cells were transduced with 20 µl NGN2 LV plus 0.5 µl Polybrene. On Day 3, cells were fed by complete media exchange with fresh mTESR Complete media. On Day 4-6, NGN2 LV transduced iPSCs were expanded and frozen in mTESR complete media supplemented with 10% DMSO. For differentiation into neural progenitor cells (NPCs), NGN2 LV transduced iPSCs were thawed as described above. On the next day (Day 1), the media was changed to N2B27 media (50:50 Neurobasal [Gibco, Cat# 21103-049] and DMEM/F-12 [Gibco, Cat# 11320033] base media supplemented with 1x B-27 without vitamin A [Gibco, Cat# 12587010], N2 [Gibco, Cat# 17502-048], GlutaMAX [Gibco, Cat# 35050], Pen/Strep [Gibco, Cat# 15140122], and 1 µg/ml doxycycline [Sigma, Cat# D9891]). On Day 2, cells were switched to N2B27 media supplemented with 1 µg/ml puromycin to select for stably transduced cells. On Day 4, cells were either detached and plated in new Matrigel-coated plates or frozen down in NPC Media supplemented with 10% DMSO. For PFF experiments, NPCs were plated in Cell Carrier-96 optically clear imaging plates (Perkin Elmer, Cat# 6005550) pre-coated with 1x Matrigel at a density of 24,000 cells per well. Cells were maintained in Complete NGN2 Induction media (N2B27 Media supplemented with 1 µg/ml doxycycline, 1 µM dbcAMP [Sigma, Cat# D0260], 200 µM Ascorbic Acid [Sigma, Cat# A4403], 10 ng/mL BDNF [Tocris, Cat# 2837], and 10 ng/ml GDNF [R&D Systems, Cat# 212-GD-050]). Wells were replenished with 50% fresh NGN2 Induction media on DIV 2, 4, and 7, at which point doxycycline was removed from the media. On DIV 10, cells were fed with 50% fresh media and pre-treated for 1 h with 0, 10, or 100 nM GNF362 followed by addition of 5 µg/ml mPFFs for 11 days. On DIV 21, cells were fixed for immunocytochemistry as described below.

### Cloning, Expression and Purification of Murine α-Synuclein Monomer

Wild type full length murine αSyn with an N-terminal 6xHis tag followed by an eXact tag (Bio-Rad; Profinity eXact Fusion-Tag System) was cloned, expressed in E. coli and purified as previously described (Weihofen et al 2019). Cell pellets were suspended in lysis buffer and lysed by a microfluidizer at 12,000 psi on ice. The lysate was clarified by centrifugation and the 6xHis-eXact-murine αSyn was captured by batch binding to Ni-NTA resin (Qiagen, Superflow). The protein-bound resin was packed into a column, washed with Ni wash buffer containing 20 mM imidazole, 0.5 mM dithiothreitol and protease inhibitors, and eluted with 250 mM imidazole in Ni wash buffer. The eluate was loaded onto a Profinity eXact affinity column (Bio-Rad) equilibrated with 100 mM Na2HPO4, pH 7.2, 500 mM sodium acetate, and washed with the same buffer until a stable baseline was obtained. Cleavage of the eXact tag was triggered by washing with 100 mM Na2HPO4, pH 8, 100 mM NaF, leading to release of the untagged protein from the column. Pooled peak fractions were further purified by size exclusion chromatography (SEC). The final purified protein was concentrated to 10 mg/mL in 10 mM Tris-HCl, pH 7.6, 50 mM NaCl, filtered through a 0.1 µm filter, and 0.2 mL aliquots were stored at -80°C. The purified αSyn was >95% pure by SDS-PAGE and had a calculated molecular weight of 14 kDa by SEC-multiangle light scattering. The purified αSyn had a hydrodynamic radius of 3.5 nm with no detectable large particles by dynamic light scattering.

### Preparation of murine α-synuclein preformed fibrils

Purified full length murine α-syn (0.2 mL) was thawed in a 37°C water bath. The protein was centrifuged with a Beckman tabletop high speed centrifuge at 600,000 g for 30 min at 4°C. The 200 µL clarified α-syn monomer solution was transferred to a 1.7 mL Eppendorf tube and fibrillar α-syn formed by continuous shaking at 1100 rpm in an Eppendorf Thermomixer at 37°C for 5 days.

### Antibodies

The following antibodies were used in conjunction with either Western blot, ELISA, or immunocytochemistry analysis as described below.

Western blotting

| Target | Host | Supplier | Cat# | Dilution |
|---|---|---|---|---|
| Phospho- α-synuclein (Ser129) | Rabbit monoclonal [EP1536Y] | Abcam | ab51253 | 1:1,000 |
| Total α-synuclein (mouse and human) | Mouse monoclonal [SYN-1, Clone 42] | BD Biosciences | 610787 | 1:1,000 |
| Total AMPK | Rabbit monoclonal [D5A2] | Cell Signaling Technology | 5831S | 1:1,000 |
| Phospho-AMPK (Thr172) | Rabbit monoclonal [D4D6D] | Cell Signaling Technology | 50081S | 1:1,000 |
| Total ATG16L1 | Rabbit monoclonal [EPR15638] | Abcam | ab187671 | 1:1,000 |
| Phospho-ATG16L1 (Ser278) | Rabbit monoclonal [EPR19016] | Abcam | ab195242 | 1:1,000 |
| GFP tag | Mouse monoclonal [3E6] | ThermoFisher Scientific (Invitrogen) | A-11120 | 1:5,000 |

| | | | | |
|---|---|---|---|---|
| Phospho-Akt (Ser473) | Rabbit monoclonal [D9E] | Cell Signaling Technologies | 4060S | 1:1,000 |
| Total Akt (pan) | Rabbit monoclonal [11E7] | Cell Signaling Technologies | 4685S | 1:1,000 |
| Phospho-mTOR (Ser2448) | Rabbit monoclonal [D9C2] | Cell Signaling Technologies | 5536S | 1:1,000 |
| Total mTOR (pan) | Rabbit monoclonal [7C10] | Cell Signaling Technologies | 2983S | 1:1,000 |
| LC3B | Rabbit polyclonal | Sigma | L7543 | 1:2,000 |
| ITPKB | Rabbit polyclonal | Proteintech | 12816-1-AP | 1:1,000 |
| β-Actin | Mouse monoclonal [C4] | Millipore | MAB1501 | 1:5,000 |
| MICU1 | Rabbit monoclonal [D4P8Q] | Cell Signaling Technologies | #12524 | 1:1,000 |
| MICU2 | Rabbit polyclonal | Abcam | ab101465 | 1:1,000 |
| MICU3 | Rabbit polyclonal | ThermoFisher Scientific (Invitrogen) | PA5-55177 | 1:1,000 |

ELISAs

| Target | Kit Type | Supplier | Cat# | Dilution |
|---|---|---|---|---|
| Phospho-AMPK (Thr172) | PathScan Phospho-AMPKα (Thr172) Chemiluminescent Sandwich ELISA | Cell Signaling Technologies | 7792C | Per manufacturer's instruction |
| Total AMPK | PathScan Total AMPKα Sandwich ELISA Kit | Cell Signaling Technologies | 7961C | Per manufacturer's instruction |

Immunocytochemistry

| Target | Host | Supplier | Cat# | Dilution |
|---|---|---|---|---|
| Phospho- α-synuclein (Ser129) | Rabbit monoclonal [EP1536Y] | Abcam | ab51253 | 1:1,000 |
| MAP2 | Chicken polyclonal | Abcam | ab92434 | 1:2,000 |
| NeuN | Mouse monoclonal [A60] | Millipore | MAB377 | 1:2,000 |
| Flag tag | Mouse monoclonal [M2] | Sigma | F3165 | 1:5,000 |

## Chemicals

The following chemicals were used to treat cells in experiments described in this manuscript.

| Name | Target | IC50 | Supplier | Cat# |
|---|---|---|---|---|
| GNF362 | ITPKB | 9 nM | Evotec | n/a |

| | | | | |
|---|---|---|---|---|
| 2-Aminoethoxy-diphenylborane (2-APB) | Inositol-1,4,5-triphosphate receptor ($IP_3R$) | 42 µM | Tocris | 1224 |
| Ruthenium Red | Mitochondrial calcium uniporter (MCU) | 15 µM | Sigma | R2751 |
| KB-R7943 | Mitochondrial calcium uniporter (MCU) | 5.5 µM | Tocris | 1244 |
| Ryanodine | Ryanodine receptor | 2 µM | Calbiochem | 15662-33-6 |
| Thapsigargin | Sarco/endoplasmic reticulum $Ca^{2+}$ ATPase (SERCA) pump | 1 nM | Tocris | 1138 |

### mCherry-GFP-LC3B HeLa cells

mCherry-GFP-LC3 cells constitutively overexpressing human LC3 protein dual-tagged with GFP (pH-sensitive) and mCherry (pH-insensitive), which allows for live-visualization of autophagosomes (GFP/mCherry co-positive spots) and lysosomes (mCherry+ only), as described previously (1, 2). The human LC3 gene was dual-tagged (N-terminus) with mCherry-GFP and cloned into a pLVX-Lenti vector backbone (Clontech), which was then used to produce lentivirus using the ViraPower HiPerform Lentiviral Expression Kit, according to manufacturer's instruction (Invitrogen, Cat# K533000). HeLa cells were transduced and stable pools were selected with 2 µg/ml of puromycin, and FACS sorted to single cells. A clonal line that showed medium expression level was used for experiments. For live-cell imaging, HeLa cells were plated in Cell Carrier-96 optically clear imaging plates (Perkin Elmer, Cat# 6005550) pre-coated with 1x Cell Attachment Factor (Gibco, Cat# S006100) at a density of 4,000 cells per well. 1 day later, adhered cells were transfected with a 2 nM Silencer Select siRNA cocktail targeting human ITPKB (Ambion, Cat# 4427038, IDs: s7629, s7630) or a non-targeting control sequence (Ambion, Cat# 4390843, Cat# 49390846) using RNAiMAX Lipofectamine (Invitrogen, Cat# 13778100) according to manufacturer's instructions. 48 h later, cells were incubated with NucBlue Hoechst 33342 (Invitrogen, Cat# R37605, 1:200 dilution) for 1 h and imaged using the Perkin Elmer Opera Phenix high-content imager, as described above. For protein analysis, HeLa cells were seeded in an uncoated 12-well plate at a density of 175,000 cells/well. Cells were transfected with 12.5 nM siRNA as above and media was changed the next day and again 48 h later at time of starvation and 100 nM Bafilomycin A1 treatment (Millipore, Cat# 508409). Starved cells were washed twice with HBSS and then starved for 4 h in HBSS (Gibco, Cat# 14025092). Cells were collected in RIPA supplemented with Halt protease/phosphatase inhibitor cocktail with EDTA. Protein concentrations were determined by BCA protein quantitation assay and samples were prepared for Western blot analysis using Bolt 4x LDS sample buffer and 10X Bolt sample reducing agent. was used to quantify the neurite length dynamics as readout for neuroprotective role of ITPKB in aged neurons.

### Incucyte live-imaging and neurite tracing analysis

Freshly dissociated cortical neurons from P15-18 neonatal mice were resuspended in NB/FBS Plating Media (Neurobasal + 10% FBS, 0.26x Glutamax, 1x Pen/Strep) and seeded in a 96-well poly-D-lysine coated microplate (Corning cat # 08-774-255) at 20,000 cells/well. To minimize cell growth edge effect, microplate was rested in a tissue culture hood at room temperature for 30min, then placed inside the 37c/5% CO2 tissue culture incubator. NB/FBS plating media was removed 2 h later and replaced with prewarmed NB/B27 feeding media (Neurobasal + 1x Glutamax + 1 x Pen/Strep + 2% B27). The microplate was then placed inside the IncuCyte ZOOM live-content imaging instrument, where phase and fluorescent images (4 images per well, 12 to 15-wells per treatment condition in a randomized manner, 10x objective) were captured every 12 h for entire length of the study.

On DIV 5, neurons were transduced with either control eGFP PHP.B AAV, ΔGSS hITPKB PHP.B AAV or FL hITPKB PHP.B AAV at 100k MOI and returned to the IncuCyte ZOOM instrument. NeuroTrack software was used to quantify the neurite length dynamics as readout for neuroprotective role of ITPKB in aged neurons.

### eQTL analysis

The MetaBrain eQTL summary statistics were derived from public and protected RNA-seq and DNA genotyping available to the scientific community and Biogen, and consists of the GTEx, AMP-AD, BrainSeq, PsychENCODE, NABEC, and TargetALS cohorts. Additionally, public RNA-seq data for brain was obtained from the European Nucleotide Archive (ENA), following genotyping on RNA-seq reads. The data was

harmonized using standard alignment using STAR to GRCh38, and FeatureCounts was used to quantify genes using GENCODE v24. QC and harmonization was performed using a standardized pipeline (3, 4). eQTL analysis focused on RNA-seq samples from cortex tissues and was limited to individuals of European descent. The eQTLs were computed and normalized using the QTL pipeline described in Westra et al. The final eQTL summary statistics were computed as a sample-size weighted meta-analysis across the included cohorts (Westra et al. 2013, PMID: 24013639).

### Human Tissues

Post mortem amygdala tissue from Braak Stage IV-VI Parkinson's disease and non-demented control donors were obtained from the Netherlands Brain Bank (NBB, Netherlands Institute for Neuroscience, Amsterdam, open access: www.brainbank.nl). Written informed consent for the use of the samples for research purposes and clinical information of donors was obtained by the NBB. Patient demographic info for all tissues analyzed is available in Supplementary Table 2.

### Postnatal AAV injections

Adeno-associated viruses (AAVs) were injected into mice on postnatal day 0 (P0). Pups were anesthetized on ice and were then injected intracerebroventricularly (i.c.v.) with 4 µl virus (1E11 GCs) mixed with fast green dye (<1% total). Mice were then warmed on a heating pad until recovered and monitored post-surgery according to IACUC approved guidelines. P0 injected pups were then injected with saline (PBS) or murine α-syn PFFs at 2 months of age (see below).

### Stereotaxic injections

At 2 months of age, animals were anesthetized with 1.5% isoflurane and stereotaxically injected into the right hemisphere with recombinant murine α-syn PFFs (2 µl of 5 mg/ml sonicated fibrils). Control animals received sterile PBS (vehicle). A 26-gauge single needle insertion (co-ordinates: AP 0.2mm, ML -2.0mm, DV -2.6mm) into the right forebrain was used to target the inoculum to the dorsal neostriatum. Injections were performed at a rate of 0.25 µl/min with the needle in place for > 5 min at each target. Animals were monitored regularly following recovery from surgery according to IACUC approved guidelines, and sacrificed 30 days post-injection as described below.

### Biochemical fractionation for *in vivo* study

For PFF injected mice, animals were anesthetized by $CO_2$ inhalation followed by cardiac perfusion with ice-cold PBS according to Biogen IACUC guidelines. Brains were removed from the skull and the cerebral cortex, hippocampus, midbrain, and striatum tissues were dissected and immediately frozen by submersion in liquid nitrogen. Tissues were stored at -80°C until day of homogenization. Frozen cortices (ipsilateral hemisphere to PFF injection site) were homogenized in 8 volumes per weight (v/w) high-salt (HS) buffer (50 mM HEPES KOH pH 7.6, 750 mM NaCl, 5 mM EDTA) supplemented with Halt protease/phosphatase inhibitor cocktail with EDTA (Pierce, Cat# 78442) using the Qiagen TissueLyser II homogenization apparatus (Cat# 85300). 600 µl total HS lysate per sample was centrifuged at 20,000 *g* for 45 min at 4°C. The supernatant ('HS fraction') was collected and then the pellet was washed by resuspension in HS buffer followed by centrifugation at 20,000 *g* for 45 min at 4°C. Washed supernatants were discarded. The pellets were then resuspended in 300 µl 1% Triton X-100 (TX) buffer (50 mM HEPES KOH pH 7.6, 750 mM NaCl, 5 mM EDTA, 1% Triton X-100 plus protease/phosphatase inhibitors), vortexed, and incubated on ice for 15 min prior to centrifugation at 20,000 *g* for 45 min at 4°C. The supernatant ('TX fraction') was collected and then the pellet was washed by resuspension in TX buffer followed by centrifugation at 20,000 *g* for 45 min at 4°C. Washed supernatants were discarded. The pellets were then resuspended in 100 µl SDS buffer (50 mM HEPES pH 7.6, 2% SDS plus protease/phosphatase inhibitors), vortexed, and incubated at RT for 30 min followed by centrifugation at 20,000 g for 45 min at 25°C. The supernatant ('SDS fraction') was collected. Protein concentration in each fraction was determined by BCA assay and analyzed by Western blotting as described above.

### Tissue processing and immunostaining

Mice were anaesthetized with CO2 and transcardially perfused with phosphate buffered saline PBS and EDTA according to Biogen IACUC guidelines. Following decapitation, brains were stored in 3% paraformaldehyde/PBS for 72 hours at 4°C. Samples were then transferred to a gradient of 15-30% sucrose/PBS prior to cryosectioning (sagittal) at 40µm and stored in cryoprotectant at -20°C until processed for tissue staining. Tissue processing and immunofluorescent staining for anti-flag, neurons, glial cells and phosphorylated α-synuclein (p129) was performed as reported previously (5). All primary antibodies were

diluted to their optimized dilutions in 0.1% Triton-X containing phosphate buffered saline (PBS): chicken microtubule associated protein family (MAP2; Abcam ab92434; 1:1000), guinea pig Neuronal Nuclei (NeuN; Sigma ABN90; 1:1000), mouse anti-FLAG (anti-FLAG; Sigma MABS1256; 1:500), rabbit Ionized calcium binding adaptor molecule 1 (Iba1; Wako 019-19741; 1:500), mouse glial fibrillary acid protein (GFAP; Abcam ab7260, 1:500), rabbit glial fibrillary acid protein (GFAP; Dako GA524, 1:500), and rabbit pS129 α-synuclein (P129; Abcam ab51253; 1:1000). All sections were also stained for DAPI (Sigma) and mounted on glass coverslips in mounting medium and stored at 4 °C until imaging.

### Imaging and cell quantification

Methodologies for imaging and counting the number of transduced cells, glial cell numbers, a P129+ cells in the cortex were adapted from those previously reported (6). In brief, a total of two sections per animal were used to quantify the percentage of transduced cells in the cortex, the number of glial cells in the cortex, and the percentage of P129+ cells in the cortex. Images were acquired on a TissueGnostics TissueFAXS SL automated slide scanner, equipped with a Zeiss AxioImager Z2 microscope, Crestoptics X-light V2 spinning disc confocal head, Lumencore Spectra 3 light source, and Hamamatsu ORCA Flash 4.0 V2+ camera. Quantitative analysis was performed on three and four-labeled fluorescent images generated by 20x confocal montage imaging of an entire sagittal mouse brain section compiled from individual images acquired using a Zeiss 20x/0.8NA Plan-Apochromat lens. The cortex was delineated as an active ROI based on neuroanatomical landmarks and with reference to the sagittal atlas of the mouse brain (Allen Brain Atlas). All slides were scanned under the same conditions for magnification, exposure time, lamp intensity and camera gain. For quantitative assessment, NeuN +, IBA1 +, GFAP + and P129+ cells from the selected ROIs were automatically detected using a custom automated image analysis software created at Biogen, and all images were batch analyzed together. Once two sections of each brain were counted, NeuN +, IBA1 +, GFAP + and P129+ cells were normalized to the area of the ROI (um2) or to the total number of DAPI+ nuclei within the ROI. Representative 20x confocal montage images were generated for each treatment group using a Zeiss AxioImager Z2 microscope and a Zeiss 20x/0.8NA Plan-Apochromat lens.

## References


1. Kimura S, Noda T, & Yoshimori T (2007) Dissection of the autophagosome maturation process by a novel reporter protein, tandem fluorescent-tagged LC3. *Autophagy* 3(5):452-460.
2. Pankiv S*, et al.* (2007) p62/SQSTM1 binds directly to Atg8/LC3 to facilitate degradation of ubiquitinated protein aggregates by autophagy. *J Biol Chem* 282(33):24131-24145.
3. Zhernakova DV*, et al.* (2017) Identification of context-dependent expression quantitative trait loci in whole blood. *Nat Genet* 49(1):139-145.
4. Ng B*, et al.* (2017) An xQTL map integrates the genetic architecture of the human brain's transcriptome and epigenome. *Nat Neurosci* 20(10):1418-1426.
5. Bantle CM*, et al.* (2019) Infection with mosquito-borne alphavirus induces selective loss of dopaminergic neurons, neuroinflammation and widespread protein aggregation. *NPJ Parkinsons Dis* 5:20.
6. Tapias V, Greenamyre JT, & Watkins SC (2013) Automated imaging system for fast quantitation of neurons, cell morphology and neurite morphometry in vivo and in vitro. *Neurobiol Dis* 54:158-168.

## Supporting Information Appendix - Figures and Tables

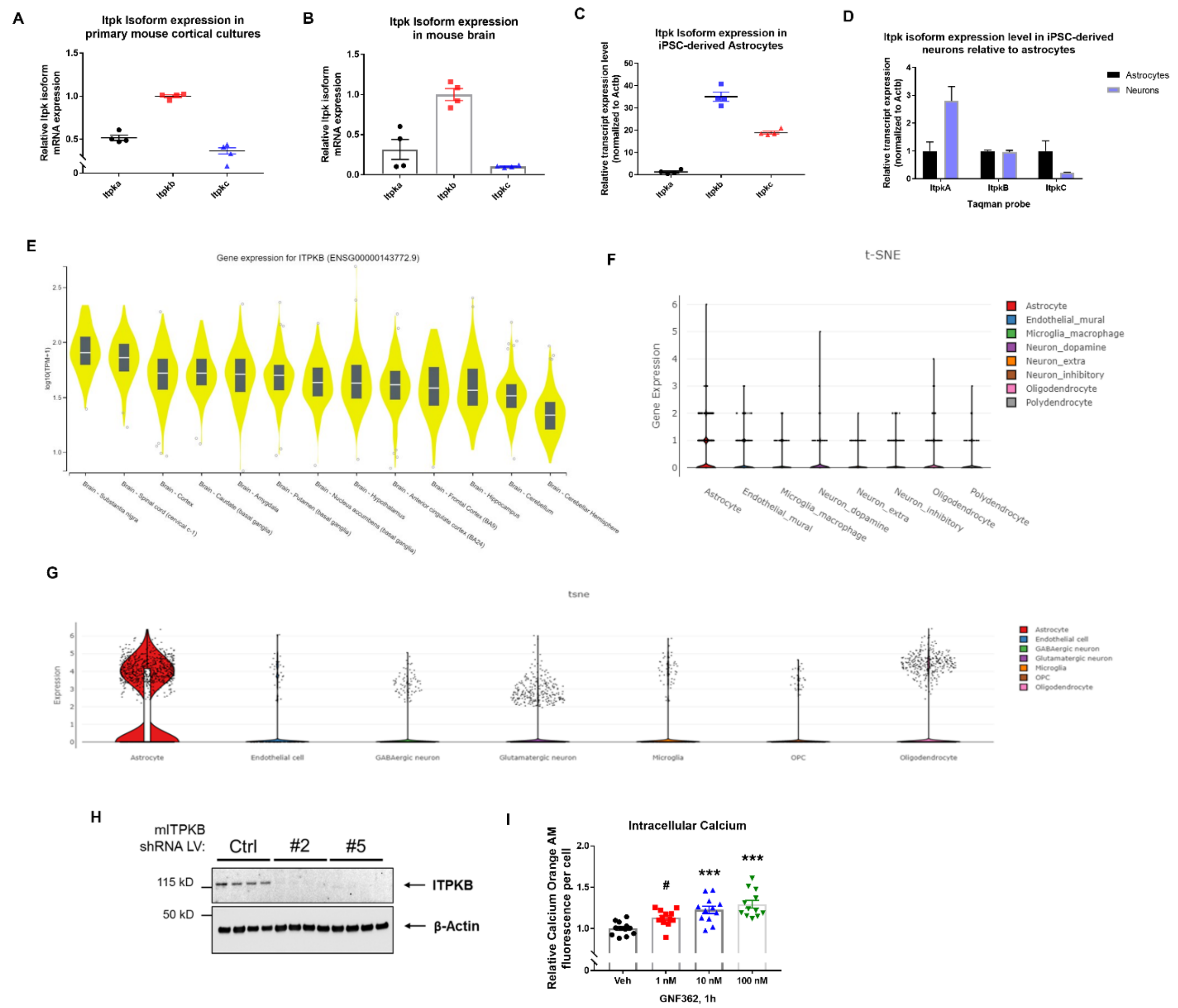


**Figure S1.** ITPKB is abundantly expressed in CNS tissues including primary mouse cortical neurons. **A-B.** Quantitative RT-PCR analysis of mRNA isolated from DIV 18 primary mouse cortical neuron cultures (A) and 2-month old C57BL/6J adult mouse whole brain tissue (B). CT values for each transcript of interest were subtracted from CT values for Actb within the same samples, and then normalized to relative expression of ItpkB equal to 1. **C-D.** Quantitative RT-PCR analysis of mRNA isolated from human astrocytes (C) differentiated from iPSCs and compared to relative transcript levels detected in human neurons (D). For each target, all values are normalized to relative transcript expressions in astrocytes equal to 1. **E.** CNS tissue expression analysis for Itpkb accessed from the Genotype-Tissue Expression (GTEx) Project on 09-10-2020 (https://www.gtexportal.org/home/gene/ITPKB) ordered from highest to lowest tissue expression. Note that Itpkb expression is enriched in CNS tissues and expressed highly in the Substantia nigra, cortex, striatum, and amygdala. **F.** Single-cell transcriptomic data from human Substantia nigra and midbrain-VTA area (https://singlecell.broadinstitute.org/single_cell/study/SCP478/single-nuclei-dataset-sn-vta-md720#study-summary) downloaded from the Broad Institute. **G.** Single nuclear RNA-Seq from human

cortex downloaded from the Broad Institute (https://singlecell.broadinstitute.org/single_cell/study/SCP371/experiment-1-all#study-visualize https://www.ncbi.nlm.nih.gov/pmc/articles/PMC6606589/). **H.** Primary mouse cortical neurons were transduced with lentiviruses (LV) expressing shRNA sequences targeting murine ITPKB (mITPKB #2 versus mITPKB #5) or a control sequence targeting firefly luciferase (Ctrl) at a multiplicity of infection (MOI) equal to 3 on DIV 5 followed by immunoblot analysis of endogenous ITPKB levels at DIV 20. Note that mITPKB shRNA sequence #2 was used for all other experiments described in this manuscript. **I.** Quantification of relative Calcium Orange AM dye fluorescence per cell in DIV 15 MCN cultures treated with GNF362 for 1 h. #p=0.0751 ***p<0.001 by 1-Way ANOVA with Tukey's post-hoc test; n=12 wells/group. Bar graphs represent mean ± SEM.

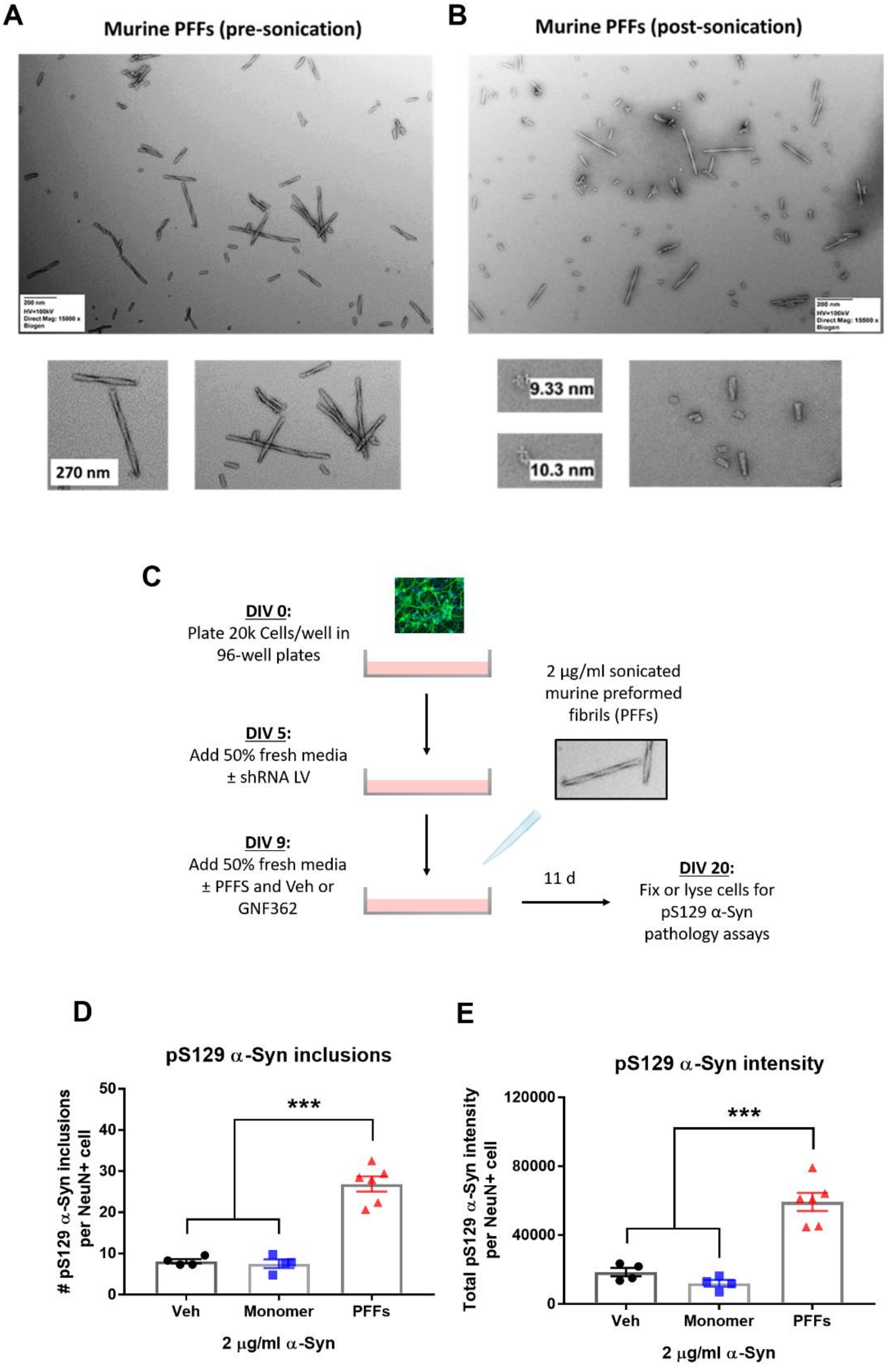


**Figure S2.** EM characterization of murine α-synuclein preformed fibrils.

**A-B.** Wild type full length murine α-Syn was cloned, expressed in E. coli, and purified as previously described (Weihofen et al. 2019) prior to aggregation into preformed fibrils (PFFs) by continuous shaking at 1100 rpm in an Eppendorf Thermomixer at 37°C for 5 days (please refer to Materials and Methods for a complete description of the PFF preparation protocol). PFFs were analyzed by electron microscopy (EM)

prior to (A) and immediately after (B) sonication using a Q Sonica water bath (1 seconds on, 1 seconds off for 1 minutes at Amplitude 50). Lower panels depict high magnification images of representative fibrils from A (left) and B (right). Scale bar = 200 nm. **C.** Diagram of experimental design for *in vitro* α-syn preformed fibril (PFF) model in primary mouse cortical neurons. Lentiviruses (LVs) were added on day in vitro (DIV) 5 at a multiplicity of infection (MOI) equal to 3 prior to treatment with 2 μg/ml murine α-syn monomer or sonicated PFFs on DIV 9. For GNF362 treated cells, neurons were pretreated with GNF362 or vehicle (DMSO) for 1 h prior to addition of PFFs with GNF362 co-treatment until DIV 20. **D-E.** Number (D) and total fluorescence intensity (E) of pS129 α-syn inclusions in cultures treated with α-syn monomer or PFFs. Note that α-syn monomer treatment did not induce pS129 α-syn pathology. ***p<0.0001 by 1-Way ANOVA with Tukey's post-hoc test; n=4-6 wells per group.

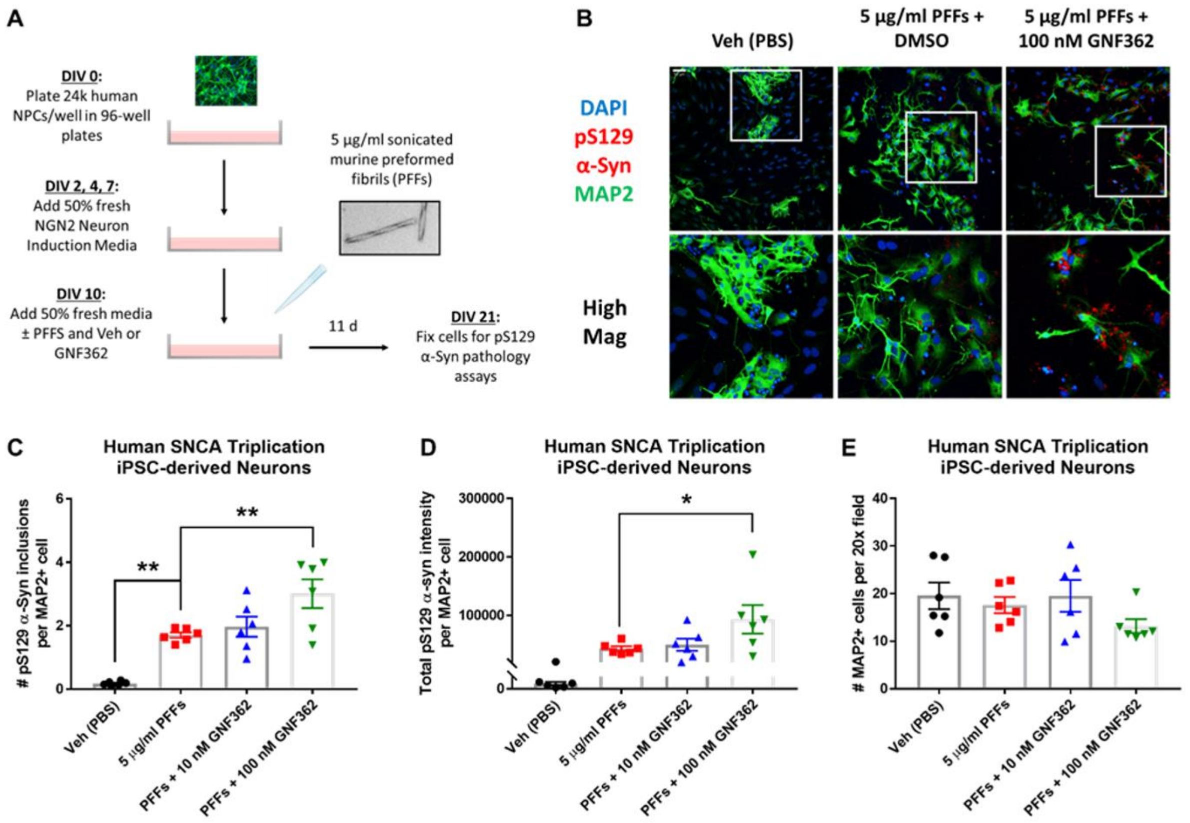


**Figure S3.** ITPKB inhibition increases α-synuclein pathology induced by preformed fibrils in human iPSC derived neurons.

**A.** Schematic of experimental design for α-syn preformed fibril (PFF) model in human iPSC derived neurons. iPSCs derived from a Parkinson's disease patient with a SNCA triplication were stably transduced with a doxycycline (dox)-inducible neuroligin 2 (NGN2) lentivirus (LV) and maintained as neural progenitor cells (NPCs) until day of experiment (DIV 0), at which point NPCs were differentiated into neurons by culturing in dox-containing NGN2 Induction Media. On DIV 10, neurons were pretreated for 1 h with GNF362 or DMSO followed by co-treatment with 5 µg/ml sonicated murine PFFs or vehicle (PBS) for 11 d. **B.** Representative images of DIV 21 NGN2 neurons immunostained for pS129 α-syn (red), the neuronal marker MAP2 (green), and DAPI. Scale bar = 50 µm. **C-D.** Quantification of the number (C) and total fluorescence intensity (D) of pS129 α-syn inclusions per MAP2-positive cell in B. *p=0.0417 **p<0.01 by 1-Way ANOVA with Dunnett's post-hoc test (vs 5 µg/ml PFF treatment alone); n=6 wells/group. **E.** Quantification of the number of MAP2-positive cells per 20x field in B. Bar graphs represent mean ± SEM.

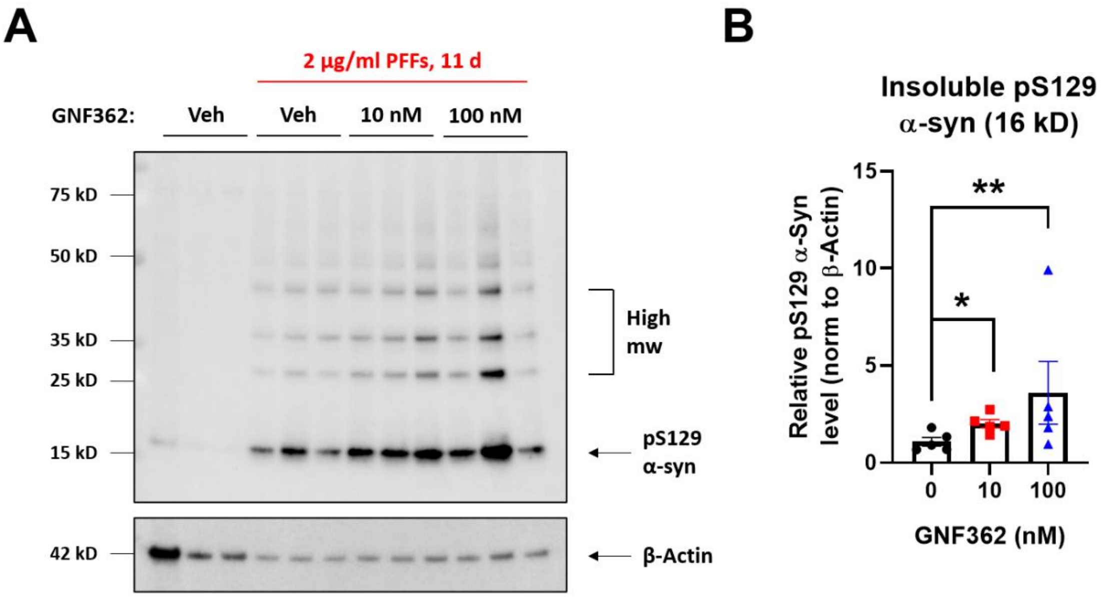


**Figure S4.** ITPKB inhibition increases α-synuclein pathology induced by preformed fibrils in mouse cortical neurons.

**A.** Representative immunoblot of pS129 α-syn in the 1% Triton X-100 insoluble fraction in mouse cortical neurons pretreated for 1 h with GNF362 or DMSO followed by co-treatment with 2 µg/ml sonicated murine PFFs or vehicle (PBS) for 11 d. **B.** Quantification of relative pS129 α-syn levels in A. *p=0.0160 **p=0.0019 by 1-Way ANOVA with Dunnett's post-hoc test; n=5/group. Bar graphs represent mean ± SEM.

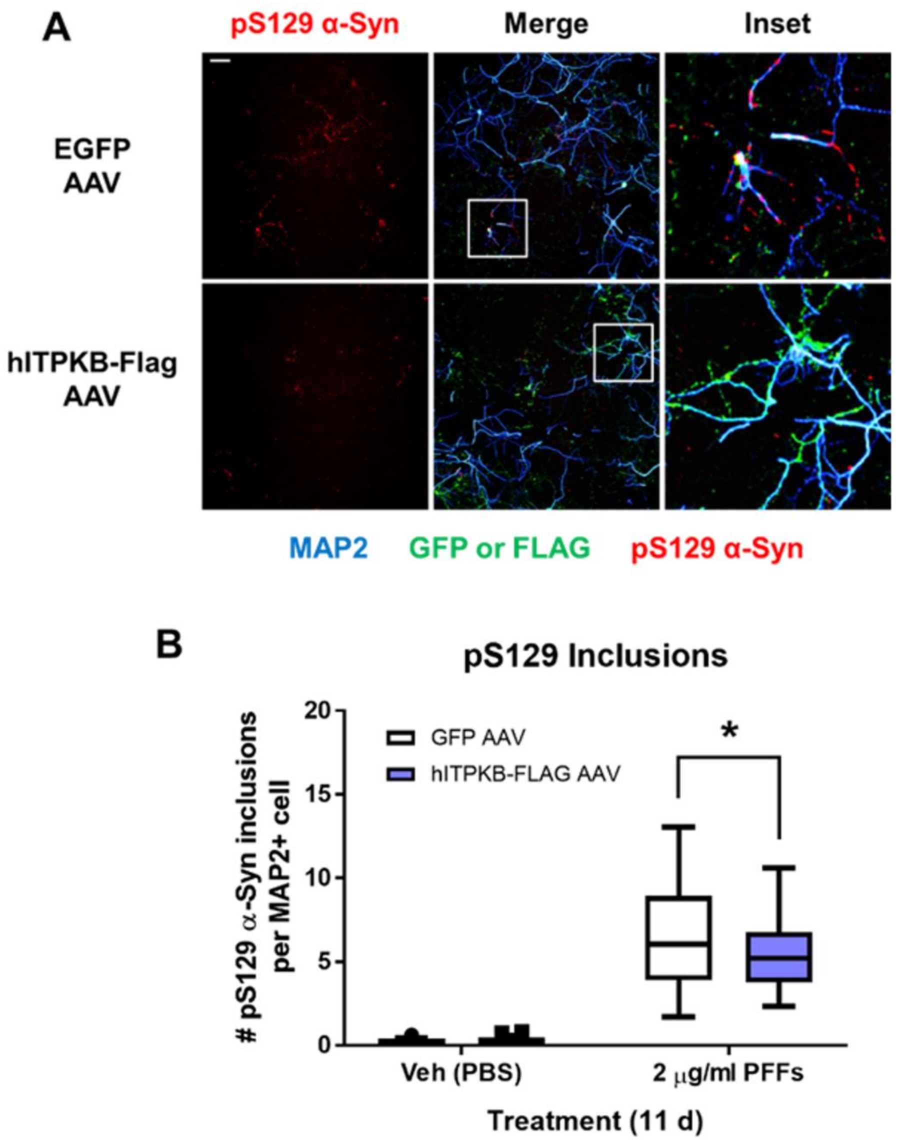


**Figure S5.** ITPKB overexpression reduces α-synuclein aggregation induced by preformed fibrils.
**A.** Representative images of neurons transduced with GFP control or hITPKB-Flag expressing AAVs treated with 2 μg/ml murine PFFs for 11 d and immunostained for pS129 α-syn (red), GFP or Flag (green), and the neuronal marker MAP2 (blue). Scale bar = 50 μm. **B.** Quantification of the number of pS129 α-syn inclusions per MAP2-positive cell in A. *p=0.0313 by 2-Way ANOVA with Sidak's post-hoc test; n=30 wells/group from 3 independent experiments. Box and whisker plots represent median ± IQR (box) from min to max (whiskers).

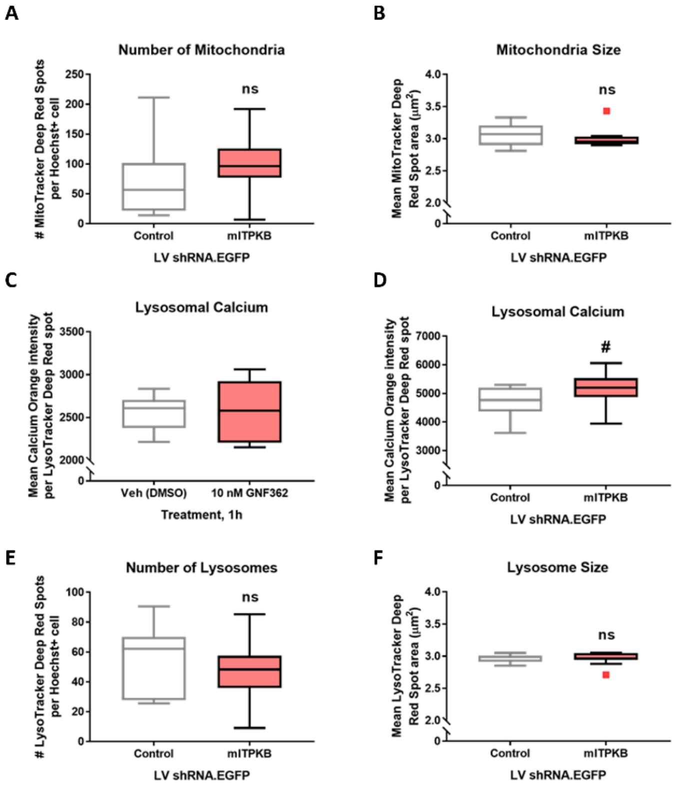


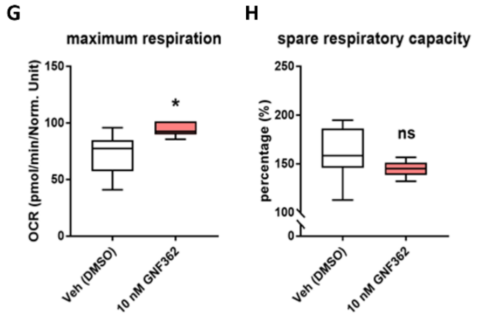


I

| Veh (DMSO) | | | 10 nM GNF362 | | |
|---|---|---|---|---|---|
| Mean | SD | N | Mean | SD | N |
| 1 | 0.1314 | 12 | 1.1665 | 0.1138 | 12 |
| 1 | 0.2299 | 6 | 1.4771 | 0.1465 | 8 |
| 1 | 0.3777 | 6 | 1.2489 | 0.6448 | 6 |

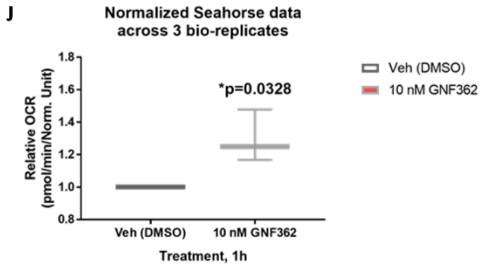


**Figure S6.** ITPKB inhibition increases mitochondrial calcium levels and respiration.
**A-B.** Quantification of number per cell (A) and size (B) of MitoTracker Deep Red-positive spots in DIV 15 Mouse cortical neurons transduced with control or mITPKB shRNA LV. **C-D.** Quantification of mean Calcium Orange AM dye fluorescence intensity in MitoTracker Deep Red-positive spots in DIV 15 neurons treated with GNF362 (C) or transduced with mITPKB shRNA LV (D). #p=0.0597 by two-tailed Mann-Whitney test; n=12 wells/group. **E-F.** Quantification of the number per cell (E) and mean size (F) of LysoTracker Deep Red-positive spots in DIV 15 neurons transduced with control or mITPKB shRNA LV. All box and whisker plots represent median ± IQR (box) from min to max (whiskers). **G-H.** Quantification of maximum oxygen consumption rate (OCR, G) and percent change in spare respiratory capacity (H) in neurons treated for 1 h with 10 nM GNF362. *p=0.0651 by two-tailed Mann-Whitney test; n=6 wells/group. **I-J.** Summary statistics (I) and mean increase (J) in baseline OCR for neurons treated for 1 h with 10 nM GNF362 across for 3 independent bioreplicate experiments. *p=0.0328 by two-tailed Mann-Whitney test; n=3 experiments.

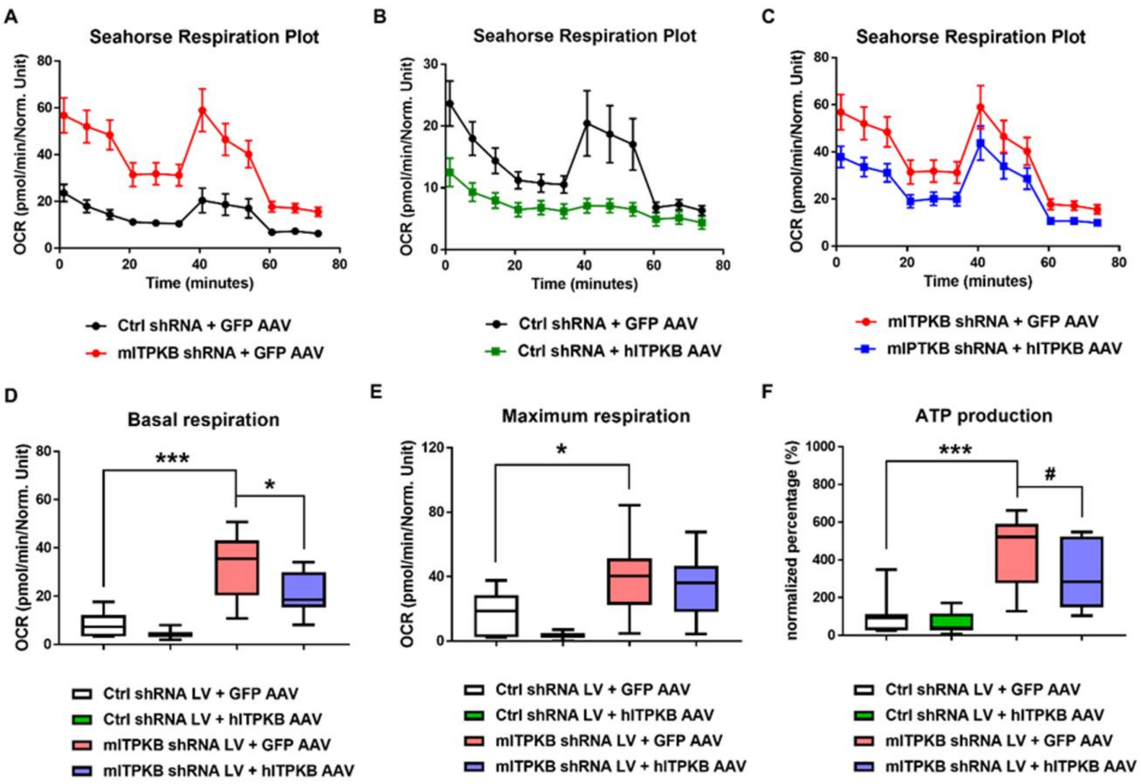


**Figure S7.** ITPKB protein level regulates mitochondrial respiration and ATP production.
Mouse cortical neurons were co-transduced on DIV 5 with control (Ctrl) or mITPKB shRNA LV and GFP or hITPKB AAV. Mitochondrial respiration was analyzed by Seahorse XF Mito Stress Test on DIV 15. Oxygen consumption rate (OCR) was measured over time starting at baseline and following sequential treatment with 1 µM oligomycin, 1.5 µM FCCP, and 0.5 µM antimycin/rotenone. **A-C.** Representative plots of OCR over time for Ctrl shRNA LV + GFP (black line) vs mITPKB shRNA LV + GFP (red line, A), GFP (black line) vs hITPKB AAV (green line) in Ctrl shRNA LV transduced cells (B), and GFP (red line) vs hITPKB AAV (blue line) in mITPKB shRNA LV transduced cells (C). All measurements were performed simultaneously within a single experiment; line graphs are plotted separately to visualize the difference between individual comparisons. **D-E.** Quantification of baseline (D) and maximum (E) OCR normalized to cell number per well from A-C. ***p<0.0001 *p=0.0313 (basal, D) *p=0.0330 (maximum, E) by 1-Way ANOVA with Tukey's post-hoc test; n=10 wells/group. **F.** Quantification of percent ATP production relative to Ctrl shRNA LV + GFP AAV from A-C. ***p<0.0001 #p=0.1822 by 1-Way ANOVA with Tukey's post-hoc test; n=10 wells/group. All box and whisker plots represent median ± IQR (box) from min to max (whiskers).

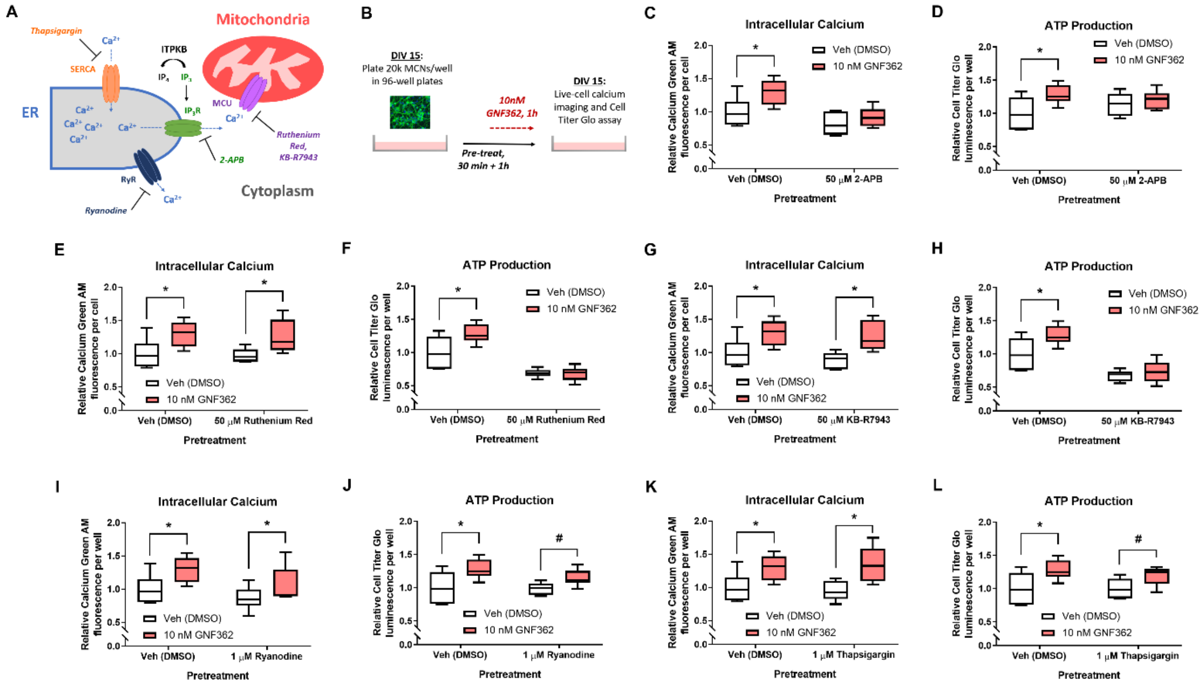

**Figure S8.** GNF362 mediated ATP production is regulated by $IP_3R$ and MCU.
**A.** Diagram of cellular receptors and channels that mediate ER calcium release and import into the inner mitochondrial matrix, including the $IP_3$ receptor ($IP_3R$, green), Ryanodine receptor (RyR, blue), sarco/endoplasmic reticulum calcium ATPase (SERCA, orange), and the mitochondrial calcium uniporter (MCU, purple). **B.** Diagram of experimental overview for C-K**.** **C-K.** Relative cellular calcium and ATP levels in neurons treated for 1 h with GNF362, as measured by Calcium Green AM dye followed by Cell Titer Glo luminescence assay for cells pre-treated with 2-APB (C-D), Ruthenium Red (E-F), KB-R7943 (G-H), Ryanodine (I-J), or Thapsigargin (K-L). *p<0.05 #p=0.10 by 2-Way ANOVA with Sidak's post-hoc test; n=6 wells/group. Note that for J-K, all measurements were performed within the same experiment. All box and whisker plots represent median ± IQR (box) from min to max (whiskers).

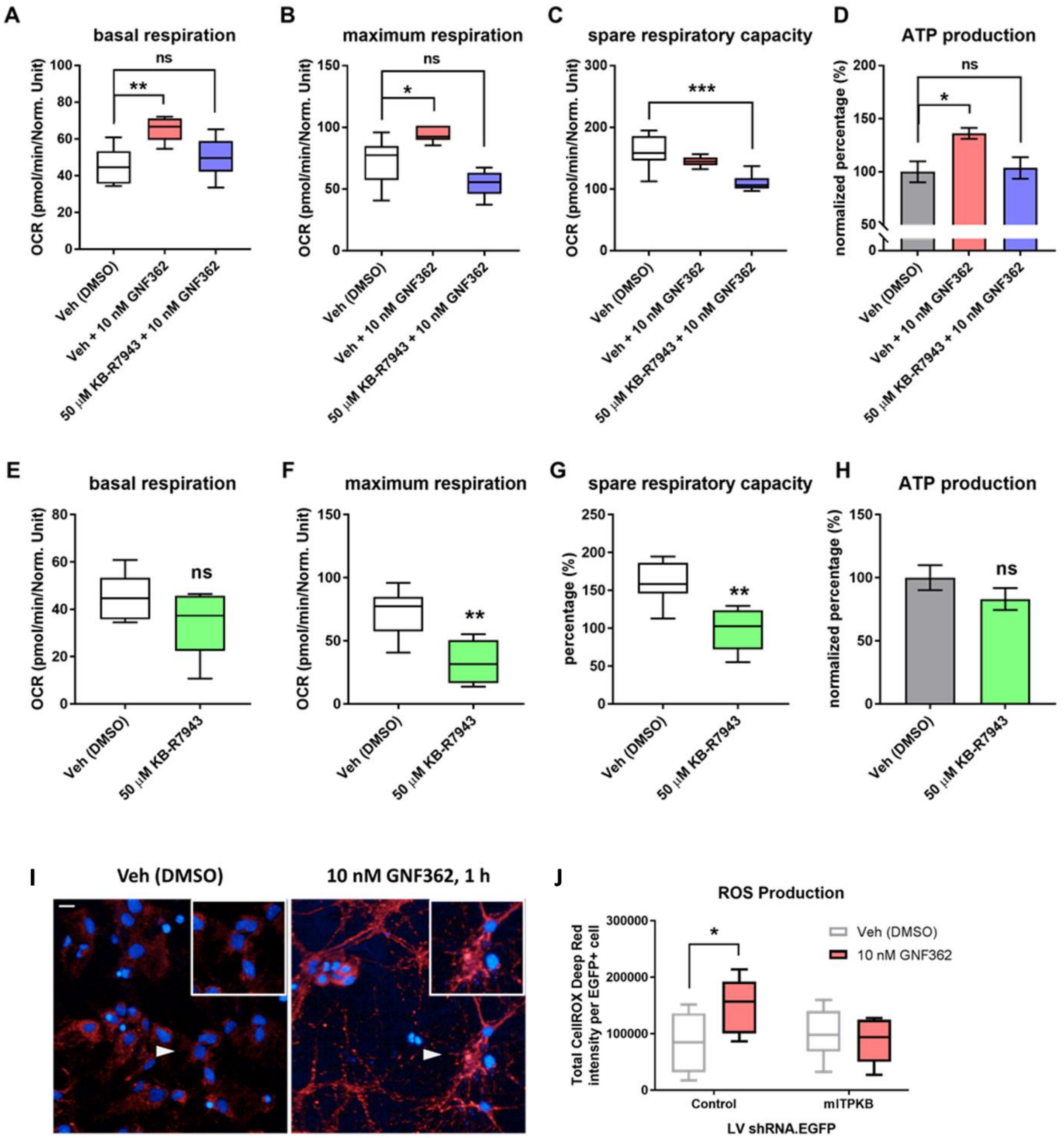


**Figure S9.** GNF362 mediated respiration is regulated by the mitochondrial calcium uniporter.
**A-D.** DIV 15 mouse cortical neurons were pre-treated with 50 µM KB-R7943 for 30 min followed by co-treatment with 10 nM GNF362 for 1 h prior to analysis of oxygen consumption rate (OCR) over time by the Seahorse XF Mito Stress Test. Baseline OCR (A), maximum OCR (B), and percent change in spare respiratory capacity (C) and ATP production (D) were normalized to cell number in each well. ***p=0.0008 **p=0.0064 *p=0.0315 (maximum respiration, B) and *p=0.0253 (ATP production, D) by 1-Way ANOVA with Tukey's post-hoc test; n=6 wells/group. **E-H.** Baseline OCR (E), maximum OCR (F), and percent change in spare respiratory capacity (G) and ATP production (H) for DIV 15 neurons treated with 50 µM KB-R7943 alone for 1.5 h prior to analysis by the Seahorse XF Mito Stress Test. **p=0.0039 (maximum respiration, F) and **p=0.0032 (spare respiratory capacity, G) by two-tailed Student's t-test; n=6 wells/group. **I.** Representative images of DIV 15 neurons incubated with Hoechst (blue) and 5 µM CellROX

Deep Red fluorescent dye (red) treated for 1 h with vehicle (DMSO) or 10 nM GNF362 prior to live-cell imaging. Insets show high-magnification images of individual cells (arrows). Scale bar = 5 µm. **J.** Quantification of total CellROX Deep Red fluorescence intensity per EGFP+ (LV transduced) cell from I. *p=0.0338 by 2-Way ANOVA with Sidak's post-hoc test (n=9 wells/group). All box and whisker plots represent median ± IQR (box) from min to max (whiskers); bar graphs represent mean ± SEM.

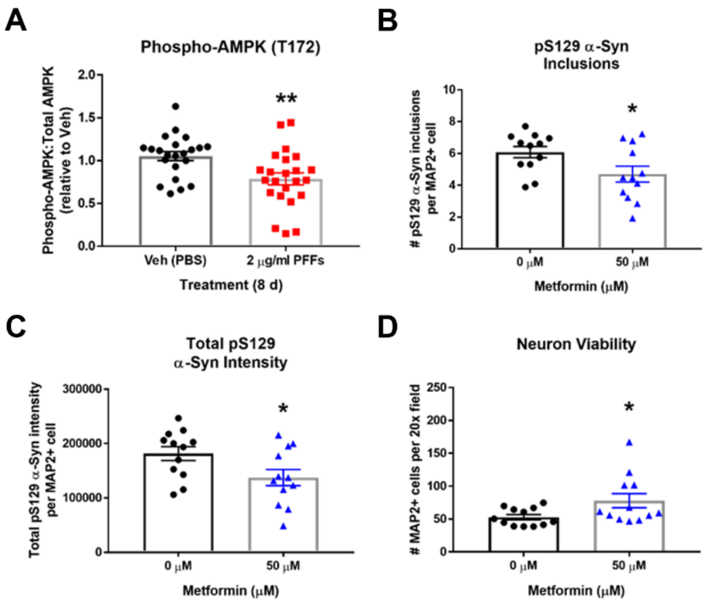


**Figure S10.** AMPK activation reduces α-syn pathology induced by PFFs.
**A.** Quantification of phospho (T172)-AMPK levels in mouse primary neurons treated with 2 μg/ml PFFs for 8 d, as determined by ELISA. Values were normalized to Total AMPK levels detected in the same samples. **p=0.0045 by unpaired Student's t-test; n=24 wells/group. **B-C.** Quantification of the number (B) and total fluorescence intensity (C) of pS129 α-syn inclusions in neurons treated with 2 μg/ml PFFs ± 50 μM Metformin for 8 d. **D.** Number of MAP2+ neurons per 20x field in neurons treated with 2 μg/ml PFFs ± 50 μM Metformin for 8 d. *p<0.05 by unpaired Student's t=test; n=12 wells/group. Bar graphs represent mean ± SEM.

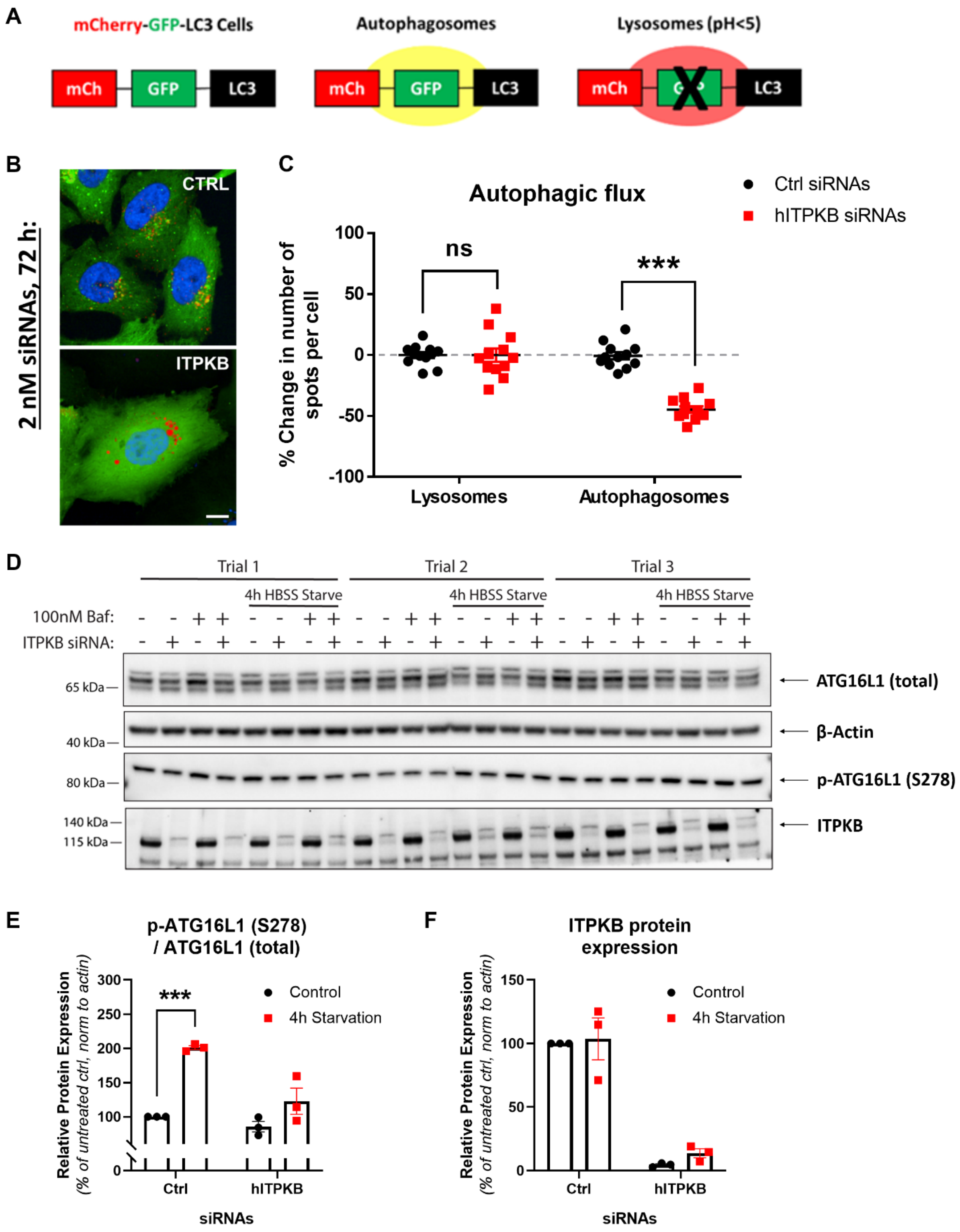


**Figure S11.** ITPKB knockdown decreases autophagosome formation and autophagic flux in mCherry-GFP-LC3B HeLa cells.

**A.** mCherry-GFP-LC3 HeLa cells stably overexpress a dual-tagged LC3 protein, which allows for visualization of autophagosomes (mCherry+/GFP+, yellow spots) and lysosomes (mCherry+/GFP-, red only) in live cells. **B.** Representative images of mCherry-GFP-LC3 HeLa cells transfected with 12.5 nM control (Ctrl) or hITPKB siRNAs and imaged 72 h post-transfection. Scale bar = 5 µm. **C.** Quantification of the percent change in number of lysosomes (mCherry+/GFP-, red only spots) and autophagosomes (mCherry+/GFP+, yellow spots) from B. ***$p<0.001$ by 2-Way ANOVA with Sidak's post-hoc test; n=12 wells/group. **D.** Representative immunoblot of mCherry-GFP-LC3B HeLa cells transfected with 12.5 pmol control (Ctrl) or hITPKB siRNAs for 72 h prior to treatment with 100 nM Bafilomycin (Baf) ± HBSS serum starvation for 4 h. **E.** Quantification of levels of ATG16L1 phosphorylated at S278 (p-ATG16L1) normalized to total ATG16L1 in D. ***$p=0.0003$ by 2-Way ANOVA with Sidak's post-hoc test; n=3 independent experiments/group. **F.** Quantification of ITPKB protein normalized to β-actin in D. Bar graphs represent mean ± SEM.

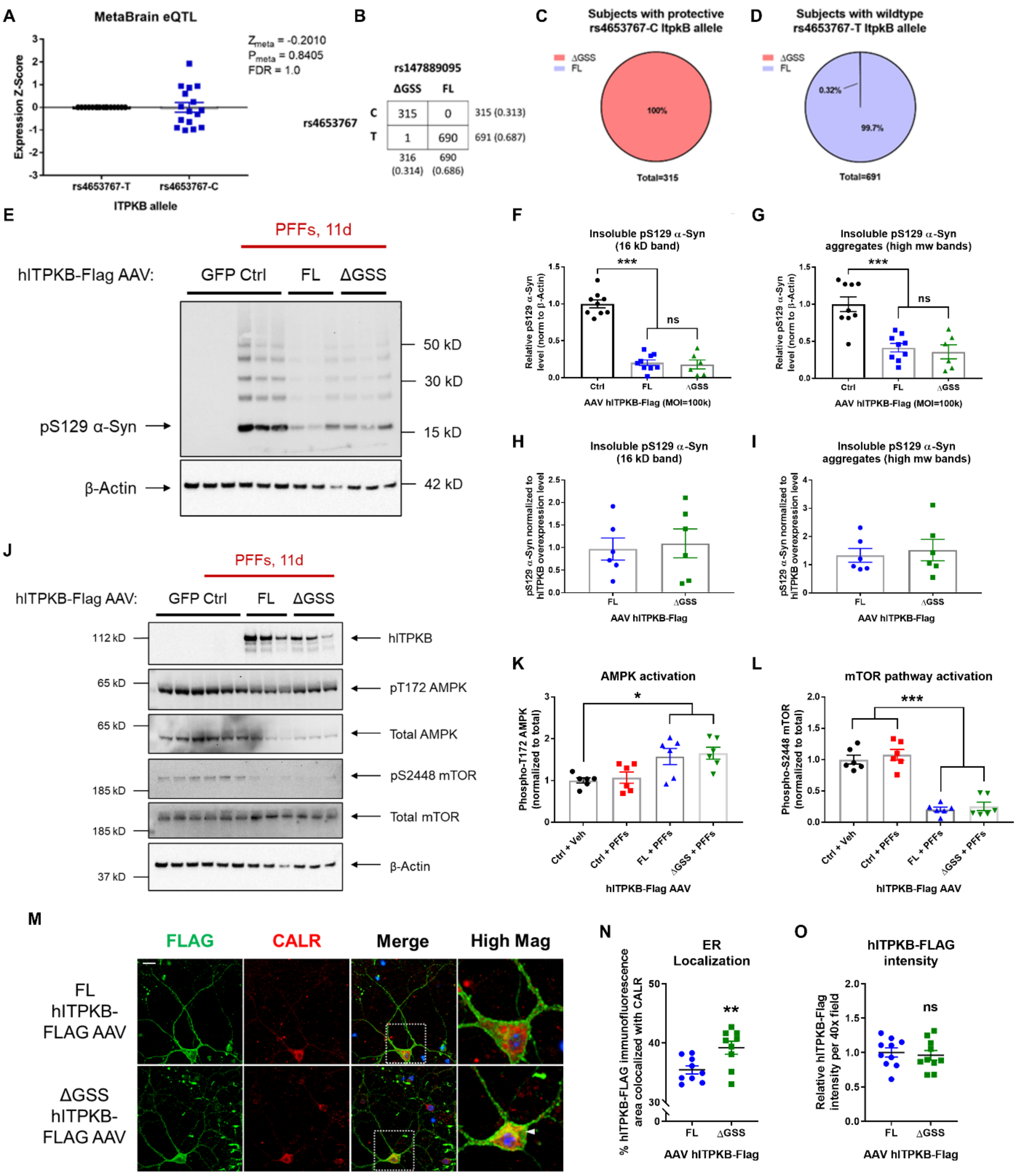


**Figure S12.** The protective rs4653767-C allele is in linkage disequilibrium with a protein coding ΔGSS deletion allele in ITPKB that increases localization to the ER.

**A.** Expression quantitative trait loci (eQTL) meta-analysis of the rs4653767-C compared to the rs4653767-T allele in whole brain tissue of 16 independent cohorts of human patients (MetaBrain eQTL). Individual data points shown represent the z-scores of mean expression changes between the rs4653767-C vs rs4653767-T allele within each study. $Z_{meta}$ = -0.2010, $P_{meta}$ = 0.8405, False Discovery Ratio (FDR) = 1.0.

**B.** Chi-Square analysis of rs4653767-C/T allele compared to rs147889095 with (ΔGSS) or without (FL) the

in-frame deletion in the protein coding sequence ($R^2$=0.9954, Chi-Sq=1001.3652, p<0.0001). **C-D.** Pie chart showing the percent of analyzed cases with the ΔGSS compared to FL ITPKB protein-coding sequence in patients with the protective rs4653767-C (C) compared to the rs4653767-T (D) allele from B. **E.** Representative immunoblot of primary mouse cortical neurons transduced with GFP Control (Ctrl) or FL vs ΔGSS hITPKB-Flag AAV (MOI = 100k) on DIV 5 followed by treatment with 2 µg/ml α-syn PFFs on DIV 9 for 11 d. **F-I.** Quantification of 16 kD (F) and high mw (G) pS129 α-syn bands in the 2% SDS soluble fraction of neurons from E. pS129 α-syn band intensity was normalized to both β-Actin (F-G) and relative to overexpression levels of hITPKB-Flag (H-I). ***p<0.001 by 1-Way ANOVA with Dunnett's post-hoc test; n=6-9 wells/group from at least 2 independent experiments. **J.** Representative immunoblot of primary mouse cortical neurons transduced with GFP Control (Ctrl) or FL vs ΔGSS hITPKB-Flag AAV (MOI = 100k) on DIV 5 followed by treatment with 2 µg/ml α-syn PFFs on DIV 9 for 11 d. **K-L.** Quantification of levels of phosphorylated (pT172) AMPK (K) and phosphorylated (pS2448) mTOR (L) normalized to total AMPK and mTOR levels in J. *p=0.0246 (FL + PFFs vs Ctrl + Veh) *p=0.0103 (ΔGSS + PFFs vs Ctrl + Veh) by 1-Way ANOVA with Dunnett's post-hoc test; n=6 wells/group from 2 independent experiments. ***p<0.001 by 1-Way ANOVA with Tukey's post-hoc test; n=6 wells/group. **M.** Representative images of DIV 18 cultures stained for Flag (green), the ER marker calreticulin (CALR, red), and DAPI (blue) following transduction with FL vs ΔGSS hITPKB-Flag AAV (MOI = 100k) on DIV 5. Scale bar = 20 µm. **N.** Quantification of the percentage of FLAG+ immunofluorescence area co-localized with CALR+ immunofluorescence in neurons from M. **p=0.0099 by unpaired Student's T-test; n=12 wells/group. **O.** Quantification of relative hITPKB-Flag immunofluorescence intensity per 40x field from M. Bar graphs represent mean ± SEM.

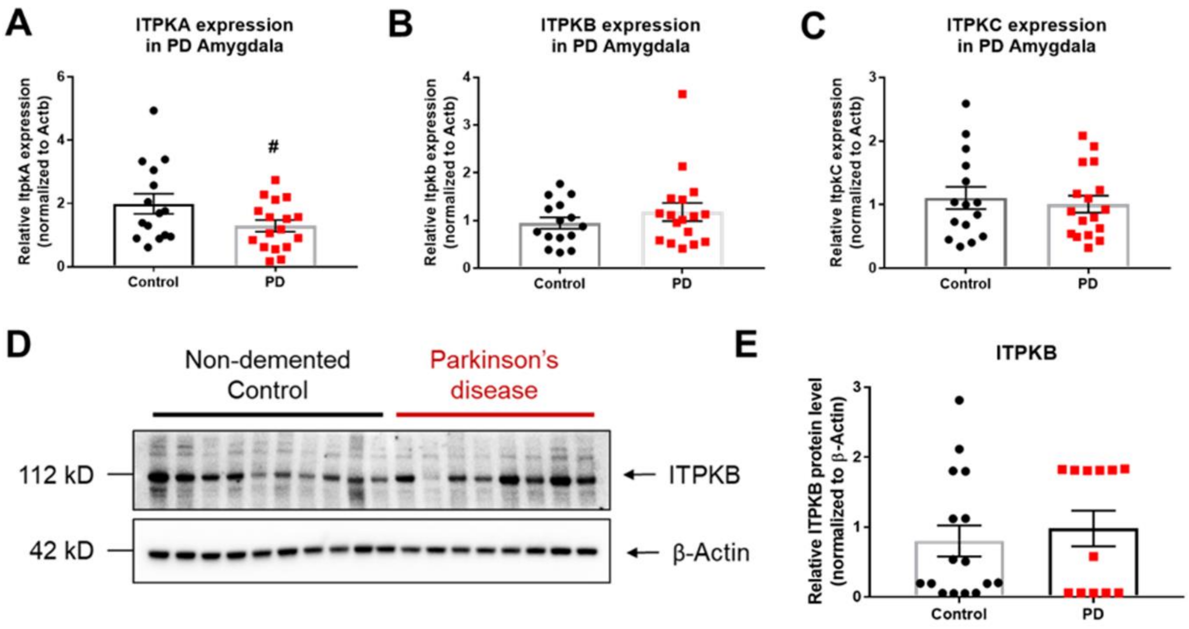


**Figure S13.** ITPKB mRNA and protein levels are not upregulated in human Parkinson's disease amygdala tissue.

**A-C.** Quantitative RT-PCR of ITPKA (A), ITPKB (B), and ITPKC (C) expression in non-demented control (Control) and Parkinson's disease (PD) human amygdala samples. #p=0.0708 by unpaired t-test with Welch's correction; n=15-17 patients/group. **D.** Representative immunoblot of ITPKB and β-Actin protein levels in Control and PD amygdala samples. **E.** Quantification of ITPKB protein levels from D. Bar graphs represent mean ± SEM.

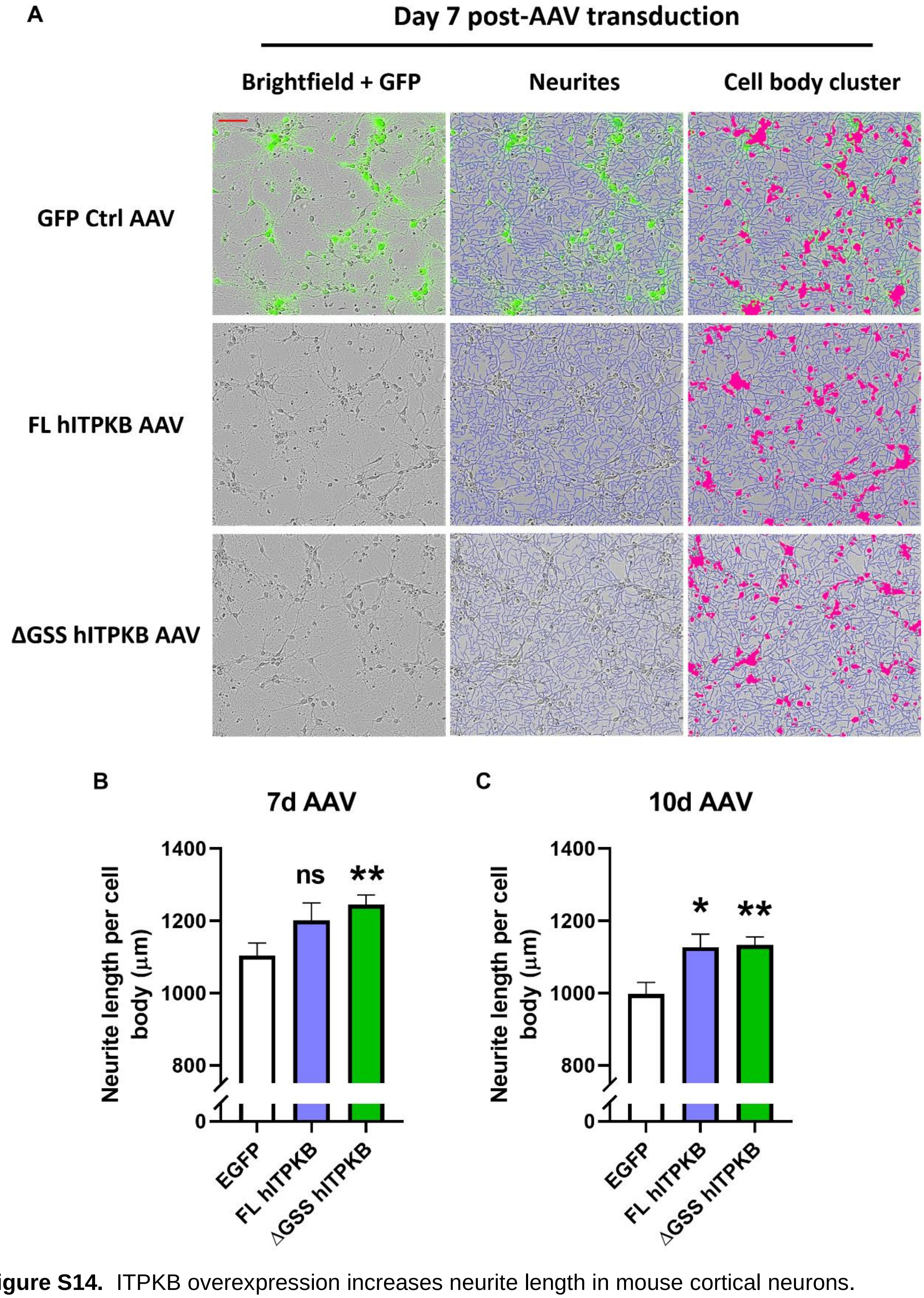


**Figure S14.** ITPKB overexpression increases neurite length in mouse cortical neurons.
**A.** Representative images of mouse primary cortical neurons transduced with control (GFP Ctrl), FL hITPKB, or ΔGSS hITPKB AAV (MOI=100k) on DIV 5 followed by live-cell imaging every 12 h using the Incucyte live-cell analysis system. Blue lines represent neurite traces (middle panels) while pink masks

represent neuronal cell bodies (right). **B-C.** Mean neurite length per neuron cell body cluster on Day 7 (B) and Day 10 (C) post-transduction with GFP Ctrl, FL hITPKB, or ΔGSS hITPKB AAVs (MOI=100k). *p=0.0300 **p<0.01 by 2-Way ANOVA with Tukey's post-hoc tests (n=15 wells/group). This experiment was repeated twice in independent cultures of primary mouse cortical neurons with different plate layouts. Bar graphs represent means ± SEM.

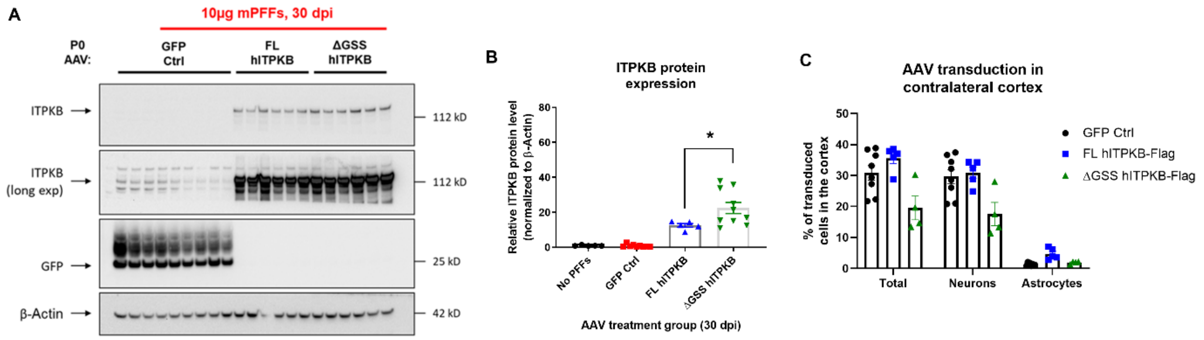


**Figure S15.** ICV injection of FL and ΔGSS hITPKB AAV leads to widespread neuronal expression in cerebral cortex.

**A.** Representative immunoblot of 1% Triton X-100 soluble lysates from ipsilateral cortex tissue 3 month after intracerebroventricular (ICV) injection of GFP, FL hITPKB, and ΔGSS hITPKB AAV in P0 mice (1E11 GCs per mouse). **B.** Relative ITPKB protein levels in each treatment group normalized to β-Actin from A. *p=0.0305 by 1-Way ANOVA with Dunnett's post-hoc test; n=5-10 mice/group. **C.** Percentage of all cells transduced for GFP Ctrl, FL hITPKB-Flag, and ΔGSS hITPKB-Flag AAV injected mice in the contralateral cortex following NeuN, GFAP, and Iba1 immunohistochemistry (IHC). EGFP fluorescence and anti-Flag immunostaining were used to identify transduced cells in the control and hITPKB AAV groups, respectively. NeuN and GFAP immunostaining were used to define neurons and astrocytes, respectively. Bar graphs represent mean ± SEM.

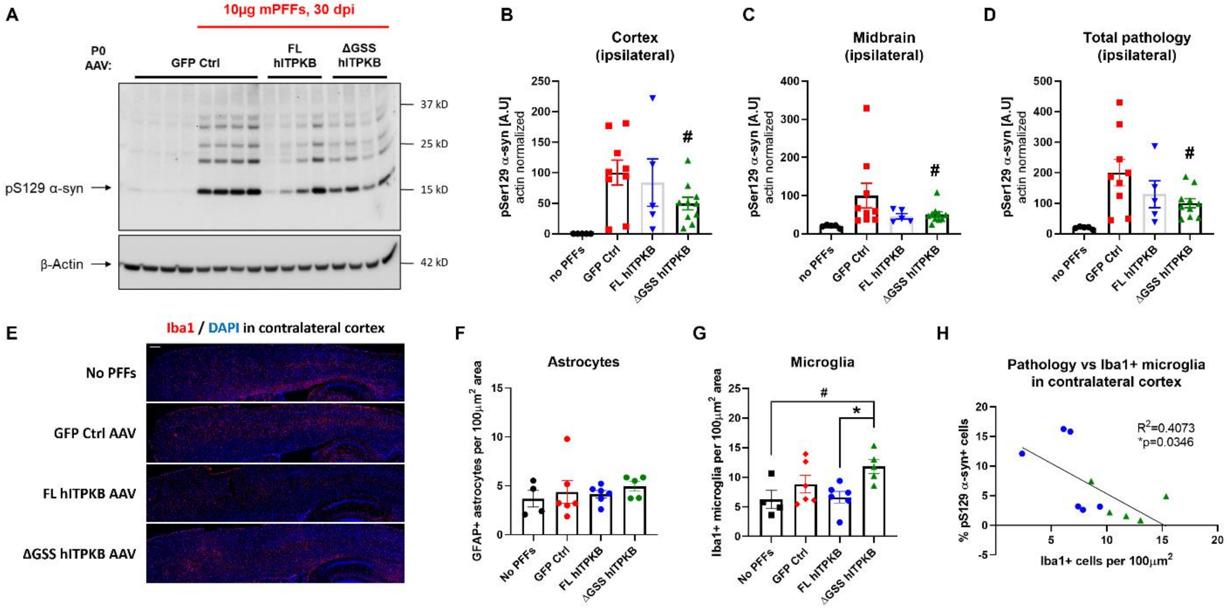


**Figure S16.** ICV injection of ΔGSS hITPKB AAV leads to an increase in Iba1+ microglia that is associated with reduced α-syn pathology in PFF-injected mice.

**A.** Representative immunoblot of pS129 α-syn pathology in the 2% SDS fraction of GFP (Ctrl), FL hITPKB, and ΔGSS hITPKB AAV injected ipsilateral cortex 30 days post-injection (dpi) with PFFs. **B-D.** Quantification of levels of pS129 α-syn pathology in the cortex (B), midbrain (C), and total (D, cortex plus midbrain) ipsilateral tissues of mice from A. #p=0.1263 (ΔGSS vs GFP Ctrl, B), #p=0.1736 (ΔGSS vs GFP Ctrl, C), and #p=0.0622 (ΔGSS vs GFP Ctrl, D) by 1-Way ANOVA with Dunnett's post-hoc test; n=5-10 mice/group. **E.** Representative images of Iba1+ (red) and DAPI+ (blue) cells in the contralateral cortex of GFP Ctrl AAV, FL hITPKB AAV, and ΔGSS hITPKB AAV injected mice. Scale bar = 50 µm. **F-G.** Number of GFAP+ astrocytes (F) and Iba1+ microglia (G) per area of contralateral cortex. #p=0.0544 *p=0.0468 by 1-Way ANOVA with Tukey's post-hoc test (n=5 mice/group). **H.** Plot of pS129 α-syn pathology vs number of Iba1+ cells within the same animals. Blue and green data points represent mice injected with FL hITPKB AAV (blue) and ΔGSS hITPKB AAV (green), respectively. *p=0.0346 by linear regression analysis ($R^2$=0.4073). Bar graphs represent means ± SEM.

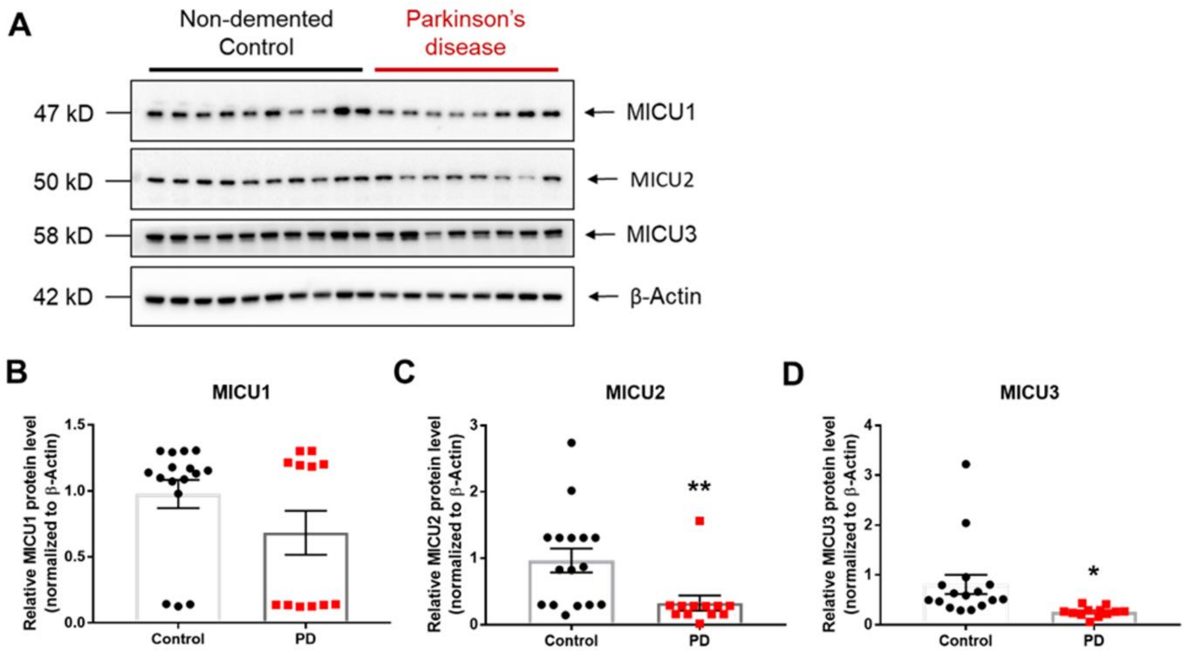


**Figure S17.** MCU regulatory subunits type 2 and 3 are downregulated in human Parkinson's disease amygdala.
**A.** Representative immunoblot of the MCU regulatory subunits MICU1, MICU2, and MICU3 in human PD and non-demented control amygdala samples. **B-D.** Quantification of levels of MICU1 (B), MICU2 (C), and MICU3 (D) from A. **p=0.0064 *p=0.0116 by unpaired t-test with Welch's correction; n=12-16 patients/group. Bar graphs represent mean ± SEM.

**Table S1.** MetaBrain eQTL analysis for rs4653767-T/C allele.
Meta eQTL analysis of rs4653767-C compared to rs4653767-T; Z-score = -0.2010, P value = 0.8405, FDR = 1.

| SNP Name | Probe Chr | Probe Center Chr Pos | Cis/Trans | SNP Type | Allele Assessed | Gene Name | Dataset | Beta | SE | Z-score |
|---|---|---|---|---|---|---|---|---|---|---|
| rs4653767 | 1 | 226685506 | cis | T/C | C | ITPKB | BipSeq-1M | -0.1790 | 0.1994 | -0.8975 |
| rs4653767 | 1 | 226685506 | cis | T/C | C | ITPKB | NABEC-H550 | -0.1557 | 0.1742 | -0.8939 |
| rs4653767 | 1 | 226685506 | cis | T/C | C | ITPKB | GTEx | -0.0860 | 0.1338 | -0.6427 |
| rs4653767 | 1 | 226685506 | cis | T/C | C | ITPKB | CMC | -0.0684 | 0.0704 | -0.9703 |
| rs4653767 | 1 | 226685506 | cis | T/C | C | ITPKB | AMPAD-ROSMAP | -0.0677 | 0.0670 | -1.0112 |
| rs4653767 | 1 | 226685506 | cis | T/C | C | ITPKB | AMPAD-MSBB-V2 | -0.0585 | 0.1053 | -0.5554 |
| rs4653767 | 1 | 226685506 | cis | T/C | C | ITPKB | CMC_HBCC_set2 | -0.0547 | 0.1786 | -0.3062 |
| rs4653767 | 1 | 226685506 | cis | T/C | C | ITPKB | TargetALS | -0.0307 | 0.1744 | -0.1763 |
| rs4653767 | 1 | 226685506 | cis | T/C | C | ITPKB | ENA | -0.0063 | 0.0473 | -0.1336 |
| rs4653767 | 1 | 226685506 | cis | T/C | C | ITPKB | UCLA-ASD | 0.0062 | 0.2071 | 0.0301 |
| rs4653767 | 1 | 226685506 | cis | T/C | C | ITPKB | LIBD_1M | 0.0440 | 0.1837 | 0.2393 |
| rs4653767 | 1 | 226685506 | cis | T/C | C | ITPKB | GVEX | 0.0857 | 0.1430 | 0.5989 |
| rs4653767 | 1 | 226685506 | cis | T/C | C | ITPKB | AMPAD-MAYO | 0.0863 | 0.1001 | 0.8621 |
| rs4653767 | 1 | 226685506 | cis | T/C | C | ITPKB | NABEC-H610 | 0.1242 | 0.1322 | 0.9397 |
| rs4653767 | 1 | 226685506 | cis | T/C | C | ITPKB | Braineac | 0.1389 | 0.1485 | 0.9350 |
| rs4653767 | 1 | 226685506 | cis | T/C | C | ITPKB | BrainGVEX-V2 | 0.2173 | 0.1131 | 1.9202 |

**Table S2.** Patient demographic info for human Parkinson’s disease immunoblot analyses.

| Patient Aliquot ID | Diagnosis | Region | Sex | Age | Braak Stage |
|---|---|---|---|---|---|
| A03 | Parkinson's disease | amygdala | f | 86 | 5 |
| A17 | Parkinson's disease | amygdala | f | 77 | 5 |
| A21 | Parkinson's disease | amygdala | f | 88 | 6 |
| A26 | Parkinson's disease | amygdala | f | 90 | 5 |
| A40 | Parkinson's disease | amygdala | m | 81 | 6 |
| A45 | Parkinson's disease | amygdala | m | 75 | 5 |
| A53 | Parkinson's disease | amygdala | f | 77 | 4 |
| A60 | Parkinson's disease | amygdala | f | 83 | 5 |
| A65 | Parkinson's disease | amygdala | f | 88 | 6 |
| A70 | Parkinson's disease | amygdala | f | 82 | 4 |
| A76 | Parkinson's disease | amygdala | f | 65 | 6 |
| A83 | Parkinson's disease | amygdala | m | 82 | 6 |
| 13 | Non-demented control | amygdala | m | 79 | 0 |
| 59 | Non-demented control | amygdala | m | 78 | 0 |
| 100 | Non-demented control | amygdala | f | 84 | 0 |
| 107 | Non-demented control | amygdala | f | 88 | 0 |
| 113 | Non-demented control | amygdala | f | 78 | 0 |
| 118 | Non-demented control | amygdala | f | 93 | 0 |
| 129 | Non-demented control | amygdala | m | 85 | 0 |
| 135 | Non-demented control | amygdala | m | 83 | 1 |
| 141 | Non-demented control | amygdala | f | 89 | 0 |
| 149 | Non-demented control | amygdala | f | 82 | 0 |
| 153 | Non-demented control | amygdala | f | 87 | 0 |
| 159 | Non-demented control | amygdala | f | 70 | 0 |
| 165 | Non-demented control | amygdala | f | 92 | 1 |
| 171 | Non-demented control | amygdala | m | 79 | 0 |
| 177 | Non-demented control | amygdala | m | 88 | 0 |
| 55B | Non-demented control | amygdala | f | 74 | 0 |
